# A Distinctive γδ T Cell Repertoire in NOD Mice Weakens Immune Regulation and Favors Diabetic Disease

**DOI:** 10.3390/biom12101406

**Published:** 2022-10-01

**Authors:** Rebecca L. O’Brien, Jennifer Matsuda, M. Kemal Aydintug, Niyun Jin, Swati Phalke, Willi K. Born

**Affiliations:** 1 Department of Immunology and Genomic Medicine, National Jewish Health, Denver, CO 80206, USA; 2 Mouse Genetics Core, National Jewish Health, Denver, CO 80206, USA; 3 National Jewish Health, Denver, CO 80206, USA; 4 Department of Immunology and Microbiology, University of Colorado Anschutz, Aurora, CO 80045, USA; 5 Autoimmunity and Inflammation Program, Hospital for Special Surgery, New York, NY 10021, USA

**Keywords:** **γδ** T cells, gamma delta T cells, type 1 diabetes, NOD mice

## Abstract

Previous studies in mice and humans suggesting that **γδ** T cells play a role in the development of type 1 diabetes have been inconsistent and contradictory. We attempted to resolve this for the type 1 diabetes-prone NOD mice by characterizing their **γδ** T cell populations, and by investigating the functional contributions of particular **γδ** T cells subsets, using V**γ**-gene targeted NOD mice. We found evidence that NOD V**γ**4+ **γδ** T cells inhibit the development of diabetes, and that the process by which they do so involves IL-17 production and/or promotion of regulatory CD4+ αβ T cells (Tregs) in the pancreatic lymph nodes. In contrast, the NOD V**γ**1+ cells promote diabetes development. Enhanced V**γ**1+ cell numbers in NOD mice, in particular those biased to produce IFN**γ**, appear to favor diabetic disease. Within NOD mice deficient in particular **γδ** T cell subsets, we noted that changes in the abundance of non-targeted T cell types also occurred, which varied depending upon the **γδ** T cells that were missing. Our results indicate that while certain **γδ** T cell subsets inhibit the development of spontaneous type 1 diabetes, others exacerbate it, and they may do so via mechanisms that include altering the levels of other T cells.

## 1. Introduction

The NOD mouse strain develops type 1 autoimmune diabetes with many similarities to the human disease, and has been instrumental in investigating genetic and immunologic contributors to the disease, common autoantigens, and therapeutic interventions [1,2,3]. However, in humans, type 1 diabetes usually arises in juveniles and is more prevalent in males than in females, whereas diabetes in NOD mice usually begins to develop during adult life, and the incidence in females is approximately double that of males. In many Specific Pathogen Free (SPF) colonies (including our own), ~75% of female NOD mice develop diabetes by 30 weeks of age, as opposed to only ~40% of male NOD mice.

The question of whether **γδ** T cells may be involved in the development of diabetes in NOD mice has been raised repeatedly, but studies addressing it often did not agree. Although three early publications implicated **γδ** T cells as protectors against this disease [4,5,6], a later study instead indicated that **γδ** T cells promote the development of NOD diabetes [7]. We reasoned that this contradiction might be explained if in reality different **γδ** T cell subsets play distinct and dissimilar roles. Indeed, in other diseases, previous studies have shown functional differences between **γδ** T cell subsets, that often correlated with the expression of particular V**γ** genes [8,9,10,11,12].

The **γδ** T cells of NOD mice, due to a thymic defect which decreases αβ T cell development but enhances that of **γδ** T cells, have been reported to be hyperproliferative and over-represented among T cells [13]. To investigate whether **γδ** T cells are connected with the unique propensity of the NOD strain to develop diabetes, we characterized **γδ** T cell populations in several tissues of the NOD mouse and compared them to those of C57BL/6 mice. In lymphoid tissues, we found several differences in NOD mice, including a greater abundance of V**γ**1+ cells relative to V**γ**4+ cells, a shift towards more IFN**γ** producing cells especially within the V**γ**1+ population in the spleen, and a deficiency of IL-17-biased cells within the V**γ**4+ population, particularly in the skin-draining lymph nodes. (Note: the Tonegawa nomenclature for the mouse V**γ** genes is used throughout this paper [14]). In addition, a higher frequency of CD8α+ V**γ**7+ cells was present among the intraepithelial lymphocytes (IELs) of the large intestine of NOD mice compared to those of B6 mice. We went on to determine the rates of spontaneous diabetes in gene-targeted NOD-background mice able to produce only **γδ** T cells of certain types or none at all, as compared to wildtype (wt) NOD controls. Whereas the diabetes incidence was reduced in NOD mice of both sexes lacking V**γ**1+ **γδ** T cells, it was accelerated in female NOD mice lacking V**γ**4+ cells. The finding that V**γ**1+ and V**γ**4+ **γδ** T cells can have different and opposing effects on the pathogenesis of NOD diabetes is consistent with similar observations in other mouse models of disease [8,9,10,15,16,17], and it could explain discrepancies in earlier reports regarding the role of **γδ** T cells in NOD diabetes [4,5,6,7]. We also explored the more immediate consequences of altering **γδ** T cells in NOD mice by inactivating TCR-**δ** or particular V**γ** genes, and found other non-targeted T cell types that were also affected, along with evidence that crosstalk occurs between **γδ** T cell subsets which regulates their levels. Overall, our results indicate that V**γ**1+ **γδ** T cells promote the development of NOD diabetes, whereas the V**γ**4+ cells protect against it, and suggest that the mechanisms involved may include subset-specific induced alterations in the levels of other T cells.

## 2. Materials and Methods (Also See Supplemental Detailed Materials and Methods)

### 2.1. Mouse Strains

Wildtype (wt) NOD/ShiLtJ (NOD) mice were purchased from Jackson Laboratories (Bar Harbor, ME, USA) and maintained in our colony. NOD.TCR**δ**-/- mice lacking all **γδ** T cells (NOD.129-T129P2-*Tcrd^tm1Mom^*) were generated by backcrossing B6.TCR**δ**-/- mice (B6.129P2-*Tcrd^tm1Mom^*/J mice, Jackson Laboratories, Bar Harbor, ME, USA) [18,19] onto the NOD background. The NOD.V**γ**1-/- strain, lacking all V**γ**1+ cells (NOD.129-TcrVg1*^tm1Car^*), was similarly generated from B6.V**γ**1-/- mice [20], as was the NOD.V**γ**4/6-/- strain, lacking both V**γ**4+ and V**γ**6+ cells (NOD.129-TcrVg4Vg6*^tm1Iku^*), from B6.V**γ**4/6-/- mice [21]. Offspring carrying the mutant allele were identified via Southern blot analysis, and homozygous strains established after at least 10 backcrosses. For NOD.V**γ**4-/- mice (NOD.129-TcrVg4*^tm1Mat^*), CRISPR/Cas9 technology was used directly in fertilized NOD oocytes to delete the entire V**γ**4 gene, by the Regional Mouse Genetics Core at National Jewish Health, so that no backcrossing was needed. All mutant NOD strains, once established, were housed in the same room in the National Jewish Biological Resource Center with unmodified NOD mice and maintained under SPF conditions. All mice were cared for following guidelines for normal and immune-deficient animals, and experiments conducted as outlined under National Jewish Health Institutional Animal Care and Use Committee approved protocol AS2504 to R.L.O.

### 2.2. Genetic Screening of Mutant Mouse Strains

After establishing backcrossed strains, genetic testing for single nucleotide polymorphisms (SNPs) known to differ between 129 strain mice and NOD mice (Jackson Laboratory, Bar Harbor, ME) showed that for NOD.TCR**δ**-/- mice, 98.5% of the SNPs were NOD-derived; only 2 SNPs within the C**δ** locus were retained that were 129-derived. For the NOD.V**γ**1-/-mice, 97.73% of the SNPs tested were NOD-derived; only 3 SNPs within the TCR**γ** locus were 129-derived. For the NOD.V**γ**4/6-/- mice, 99.24% of the SNPs tested were NOD-derived; only 1 SNP within the TCR**γ** locus was 129-derived.

### 2.3. Diabetes Monitoring

After reaching 12 weeks of age, mice were tested weekly for blood glucose levels, using glucose test strips and a glucose meter (Clarity Diagnostics, VWR, Radnor, PA, USA) to assay a drop of blood from the tail vein. A glucose level of 250 mg/dL (14 mM) or higher twice consecutively within one week was considered diagnostic of diabetes. Mice were euthanized within 2 weeks of first testing positive for diabetes, or sooner if obviously ill, or after reaching 30 weeks of age if they failed to develop diabetes previously. To examine mice with recent-onset diabetes, mice were euthanized within 1 week of first testing positive for diabetes and used as tissue sources for experiments.

### 2.4. Cell Preparation

Freshly isolated unfractionated spleen and lymph node cells from individual mice were prepared as previously described [22]. The cells from 2 pancreatic lymph nodes, and from 4 skin-draining lymph nodes (2 inguinal and 2 axillary), were pooled for individual mice. Intestinal intraepithelial lymphocytes (IEL) were prepared from the colon of each mouse, based on a previously published protocol [23].

### 2.5. Flow Cytometry

Cell surface staining on freshly prepared unfractionated lymphocytes was carried out as previously described [22]. For intracellular cytokine staining, lymphocytes were first nonspecifically activated by culturing them in medium containing Brefeldin A (10 μg/mL), PMA (75 ng/mL) and ionomycin (1.6 μg/mL), for 4–5 h at 37 °C in air containing ~10% CO_2_. For transcription factor analysis, nuclear staining was carried out using freshly isolated unstimulated cells with eBioscience Permeabilization and Fixation/Permeabilization buffers as recommended by the manufacturer (ThermoFisher Scientific, Waltham, MA, USA). Samples were analyzed on an LSRII flow cytometer (Becton Dickinson Biosciences, Franklin Lakes, NJ, USA) and the FCS files processed using FlowJo 9.9 software (Becton Dickinson Biosciences, Franklin Lakes, NJ, USA).

### 2.6. Statistics

To determine diabetes incidence in each strain, a total of 38-46 mice per group were tested weekly for diabetes from 13 to 30 weeks of age. Survival curves were generated showing the percent **t**hat had developed type 1 diabetes by each time point, and were analyzed using GraphPad Prism 6 software (Dotmatics, Boston, MA, USA); significant differences were determined by the Log-rank (Mantel-Cox) test. For bar graphs, results from 3–14 mice per group were compared unless otherwise noted in the figure legends, and significant differences determined using the Student t-test. Significant differences are denoted in the figures as: * *p* < 0.05, ** *p* < 0.01, *** *p* < 0.001, and **** *p* < 0.0001.

## 3. Results

### 3.1. The γδ TCR Repertoire of NOD Mice Is Biased in Favor of Vγ1+ Cells

**Figure 1:**
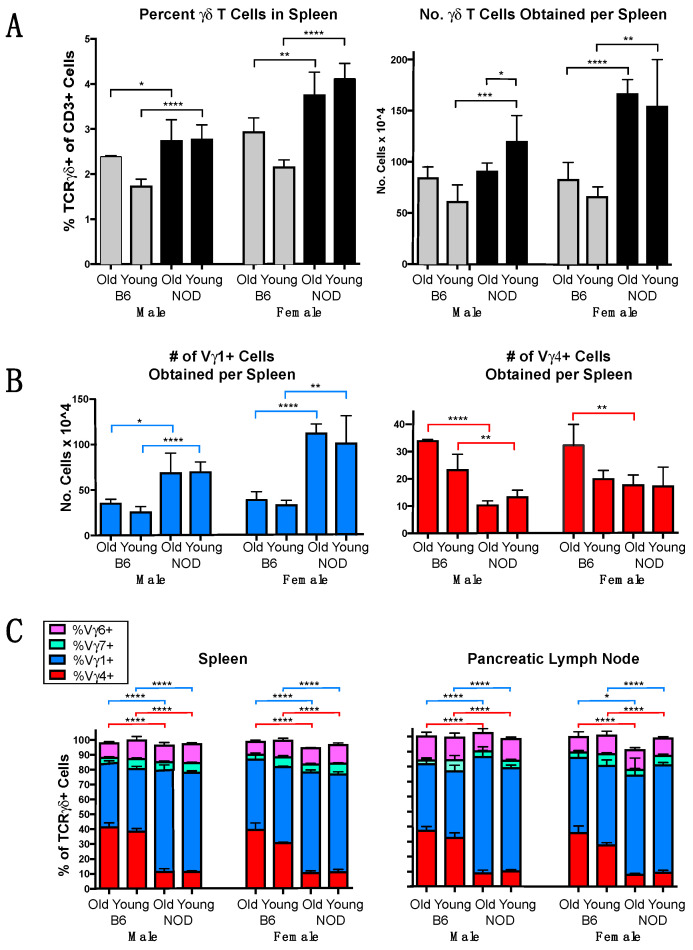
The **γδ** T cell repertoire in lymphoid organs of NOD mice compared to that of C57BL/6 (B6) mice. **γδ** T cells from individual NOD vs. B6 mice were characterized by flow cytometry, and compared within age and sex-matched groups. Young mice were between 7–10 weeks of age, older mice were between 12–34 weeks of age. For each group, samples from 4–12 mice were analyzed. (**A**). The mean percent of **γδ** T cells in spleen (left panel) and average number of **γδ** T cell obtained per spleen (right panel). (**B**). The average number of V**γ**1+ (left) and V**γ**4+ cells (right) recovered from each spleen. (**C**). The mean percentage of each V**γ**-defined **γδ** T cell subset is shown as a fraction of all **γδ** TCR+ cells, in spleen (left) and pancreatic lymph node (right). Here, and in subsequent figures, sgnifcant differences are designated as: * *p* < 0.05, ** *p* < 0.01, *** *p* < 0.001, and **** *p* < 0.0001.

The **γδ** T cells were previously shown to be comparatively abundant in NOD mice, although their levels dropped after they developed diabetes [6]. We characterized the **γδ** T cells present in NOD mice and compared them with those of C57BL/6 mice, the strain in which **γδ** T cells have been most frequently studied. Because female mice often carry greater numbers of lymphocytes than males, and levels of some lymphocytes (e.g.**,** CD4+ Tregs and memory CD8 cells) have been shown to vary with age, we compared sex-matched mice within 2 age groups, young (7–10 weeks of age) and old (12–34 weeks of age). In the spleen, **γδ** T cells were more common in NOD mice than in B6 mice for all groups (Figure 1A left), and the differences were highly significant among younger mice of both sexes. NOD mice also had higher total numbers of splenic **γδ** T cells (Figure 1A right) in all groups except for older males. In addition, greater numbers of V**γ**1+ cells were recovered from the spleens of NOD mice for both sexes and age groups, with 2–3 times as many in NOD spleens than those of matched B6 controls (Figure 1B, left). Conversely, the numbers of V**γ**4+ cells obtained from spleens were lower in NOD mice for all groups except for young females (Figure 1B, right). We also examined the V**γ** repertoire in the spleen and pancreatic lymph nodes (Figure 1C), using monoclonal antibodies specific for V**γ**1 [24], both V**γ**1 and 2 [25], V**γ**4 [26], V**γ**5 [27], and V**γ**7 [28]. An antibody specific for mouse V**γ**6 was recently reported [29], but was not available for this work, so V**γ**6+ cells were instead approximated as the calculated percent of CD3+ cells that stained **γδ** TCR+ but negative for all of the other 5 V**γ**s - specifically for V**γ**1 and 2, V**γ**4, V**γ**5, and V**γ**7 (Note: the V**γ**3 gene is missing or essentially nonfunctional in all mouse strains so far examined [25,30]). This revealed a major difference between the two strains in the percentage of **γδ** T cells expressing V**γ**4. Whereas B6 mice of both sexes and age-groups had substantial V**γ**4+ subsets comprising 30-40% of the **γδ** T cells in both spleen and pancreatic lymph node, in NOD mice, the V**γ**4+ fraction comprised only 10-15% (Figure 1C), and the vast majority were instead V**γ**1+ (70-80%, Figure 1C). The differences were highly significant within all sex- and age-matched groups for both tissues. V**γ**7+ cells and V**γ**6+ cells were minor subsets of comparable prominence in the spleens and pancreatic lymph nodes of all groups, ranging from ~3–7% for V**γ**7+ cells and ~9–12% V**γ**6+ cells (Figure 1C), whereas V**γ**+ cells, a subset that normally resides almost exclusively in the epidermis [31,32], were as expected absent or barely detected (not shown). **γδ** T cells expressing V**γ**2 also appear to be very rare or absent in these tissues, because the percentage that stained with an antibody specific for both V**γ**1 and the closely related V**γ**2 gene product (clone 4B2.9 [25]) differed by an average of less than 3% from the percentage detected with a V**γ**1-specific antibody (clone 2.11 [24]), which did not rise above the experimental variance of the samples (data not shown). Overall, the data showed a clear bias in the NOD **γδ** TCR repertoire towards V**γ**1+ and against V**γ**4+ cells, which in contrast constitute nearly equally predominant subsets in the spleen and pancreatic lymph nodes of B6 mice, whereas no major differences were seen for the other **γδ** T cells subsets.

### 3.2. Vγ1+ and Vγ4+ γδ T Cell Subsets in NOD Mice Differ from Those in B6 Mice in Cellular Composition and Functional Bias

Within the V**γ**1+ subset, a functionally distinct subpopulation has been previously described. Unlike the other V**γ**1+ cells, which co-express any of a variety of V**δ**s and when activated are biased to produce IFN**γ**, those within this subpopulation usually co-express V**δ**6.3 with a particular reading frame of D**δ**2, and evince NKT cell-like properties, which include expression of the transcription factor PLZF, and a bias when activated to produce IL-4 instead of or as well as IFN**γ** [33,34,35]. Within the V**γ**4+ subset, on the other hand, an IL-17 biased subpopulation has been described that tends to express high levels of CD44, is often CCR6+, but is negative for the TNFR superfamily member CD27 [16,36,37,38,39,40,41]. The expression of certain V**δ** genes along with V**γ**4 has also been associated with a bias to produce IL-17 [16,41,42]. In contrast, V**γ**4+ cells that produce IFN**γ** when nonspecifically stimulated are generally positive for CD27 expression [43].

**Figure 2:**
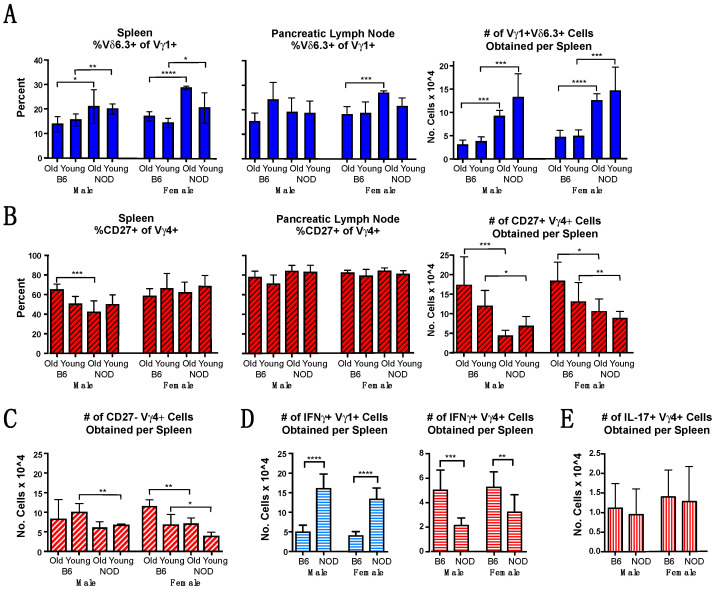
Subpopulations within the V**γ**1+ and V**γ**4+ subsets in NOD vs. B6 mice. For each group in (**A**–**C**), samples from 4–12 individual mice were analyzed by flow cytometry. For (**D**–**E**), spleen cells were nonspecifically stimulated with PMA/ionomycin before staining for flow cytometry; samples from 6–13 mice were analyzed for each group. (**A**). Mean percent of V**γ**1+ cells co-expressing V**δ**6.3 in spleen (left) and pancreatic lymph nodes (center), and average number of V**γ**1V**δ**6.3 cells recovered from spleens in each group (right). (**B**). Mean percent of V**γ**4+ cells expressing CD27 in spleen (left) and pancreatic lymph nodes (center), and average number of CD27+ V**γ**4+ cells recovered from spleen (right). (**C**). Average number of CD27- V**γ**4+ cells recovered from each spleen. (**D**). Numbers of spleen cells that expressed IFN**γ** for each sample, within V**γ**1+ cells (left), and V**γ**4+ cells (right). (**E**). Number of V**γ**4+ spleen cells expressing IL-17 for each sample. * *p* < 0.05, ** *p* < 0.01, *** *p* < 0.001, and **** *p* < 0.0001.

To compare functional potential, we went on to examine these subpopulations within the V**γ**1+ and V**γ**4+ subsets. We estimated the prevalence of the NKT-like V**γ**1+ subpopulation by testing for V**δ**6.3 co-expression among V**γ**1+ cells in NOD vs. B6 mice. In the spleen, a higher percentage of V**γ**1+ cells co-expressing Vδ6.3 was evident in all groups of NOD mice (Figure 2A left), whereas in pancreatic lymph nodes, only the older NOD females were higher. Importantly, the greater frequency in NOD mice of V**γ**1Vδ6.3+ cells combined with the higher percentage of **γδ** T cells and of V**γ**1+ cells in particular resulted in much higher absolute numbers of V**γ**1V**δ**6.3+ cells in NOD vs. B6 spleens for all groups (Figure 2A right). We also examined the proportion of V**γ**4+ cells likely to be IFN**γ-**biased by looking at the percentage that co-expressed CD27, and found that roughly half of the V**γ**4+ cells were CD27+ in all groups (Figure 2B left). These cells were somewhat rarer in the spleens of older NOD males, but there was no evident difference among females. In the pancreatic lymph nodes, no differences between groups were seen and the CD27+ fraction of V**γ**4+ cells was uniformly higher, averaging at about 80% in all groups (Figure 2B center). However, because NOD mice had fewer numbers of total V**γ**4+ spleen cells, all groups of NOD mice had smaller absolute numbers of CD27+ presumably IFN**γ**-biased V**γ**4+ spleen cells than their B6 counterparts (Figure 2B right). The older NOD males and both groups of NOD females also had slightly fewer CD27- and presumably IL-17-biased V**γ**4+ cells (Figure 2C).

We also directly tested whether the cytokine biases of V**γ**1+ and V**γ**4+ **γδ** T cells in NOD mice differ from those of B6 mice, using intracellular cytokine staining to compare the absolute numbers of spleen cells within the V**γ**1+ and V**γ**4+ subsets that produced IFN**γ** or IL-17 when nonspecifically stimulated. Though considerable variation in the percentages among individual animals was found, this did not depend upon the age of the mouse (data not shown), so for this analysis, young and old mice were analyzed together in a single group. Greater numbers of IFN**γ**-biased V**γ**1+ cells were obtained for both male and female NOD mice, exceeding those obtained from matched B6 controls by 3–4-fold (Figure 2D, left panel). Although IFN**γ**-biased V**γ**4+ cells were in contrast somewhat less numerous in NOD spleens (Figure 2, right panel), because of their low total V**γ**4+ cell numbers, the overall numbers of IFN**γ**-biased **γδ** T cells obtained from NOD mice still substantially exceed those from B6 mice. No differences were evident in the numbers of IL-17-biased V**γ**4+ spleen cells were obtained (Figure 2E), consistent with our staining results for numbers of V**γ**4+ CD27- cells, which predicted only minor differences (see Figure 2C).

We also compared the V**γ**1+ and V**γ**4+ cells in NOD vs. B6 mice for their bias to produce IL-2, and found that NOD mice had a lower average frequency of IL-2-biased cells in both subsets, compared to B6 controls (Figure S1, triangles). A lower frequency of IFN**γ**-producing V**γ**4+ cells was also evident in both male and female NOD mice. The two strains had similar average frequencies for V**γ**1+ cells producing IFN**γ** and for V**γ**4+ cells producing IL-17, whereas V**γ**1+ cells that produced IL-17 were virtually absent in both strains (Figure S1). Thus, overall, when compared with B6 mice, NOD mice exhibited distinct cytokine biases in their splenic V**γ**1+ and V**γ**4+ **γδ** T cells, and carried more IFN**γ**+ V**γ**1+ cells, fewer IL-2+ cells of either subset, and fewer IFN**γ** biased V**γ**4+ cells than B6 mice. However, both strains carried comparable numbers and proportions of IL-17-biased V**γ**4+ spleen cells.

### 3.3. A Conspicuous Deficiency in NOD Mice of Vγ4+ IL-17-Biased Cells in Skin-Draining Lymph Nodes

**Figure 3:**
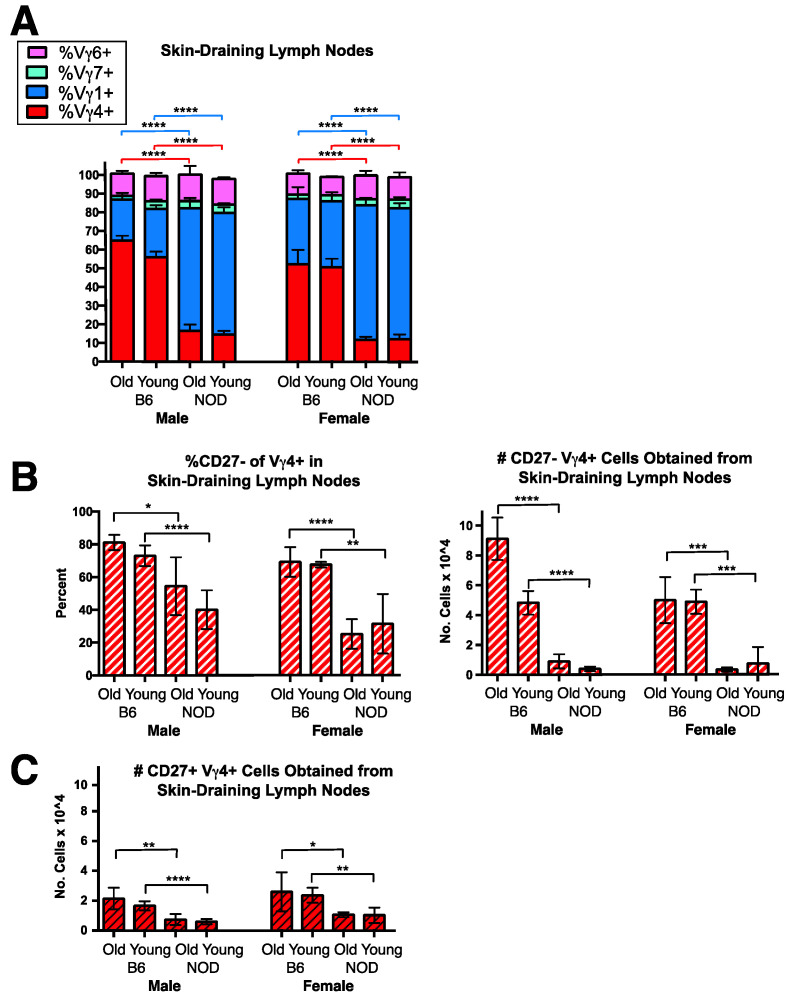
The **γδ** T cell repertoire in skin-draining lymph nodes of NOD and B6 mice. Results of samples from 4–12 individual mice were analyzed by flow cytometry for each group. (**A**). The mean percentage of each V**γ**-defined **γδ** T cell subset is shown for each group, as a fraction of all **γδ** TCR+ cells within skin-draining lymph nodes. (**B**). The mean percentage of V**γ**4+ cells that failed to stain with CD27 is shown for each group as a fraction of all V**γ**4+ cells (left), and the average number of CD27- V**γ**4+ cells obtained per mouse from the 4 pooled skin-draining lymph nodes (right). (**C**). Average numbers of CD27+ V**γ**4+ cells obtained from 4 pooled skin-draining lymph nodes. * *p* < 0.05, ** *p* < 0.01, *** *p* < 0.001, and **** *p* < 0.0001.

Because murine V**γ**4+ cells biased to produce IL-17 are known to be particularly abundant in the dermis and in skin-draining lymph nodes [16,36,40,44,45,46], we examined the status of these cells in the inguinal and axillary skin-draining lymph nodes of NOD mice (Figure 3). Consistent with published data [36,40,41], V**γ**4+ cells in the skin-draining lymph nodes of B6 mice were clearly more abundant than in spleen and in fact here comprised the major **γδ** T cell subset, surpassing the V**γ**1+ cells. However, in NOD mice, V**γ**4+ **γδ** T cells in the skin draining lymph nodes remained a minor subset (Figure 3A), averaging only 10–15% of the total **γδ** T cells. The prevalence of the other two **γδ** T cells subsets was comparable to that seen in spleen in both strains, averaging at 10–15% for V**γ**6+ cells and at 3–7% for V**γ**7+ cells. As expected [45,47], the V**γ**4+ subset in B6 skin-draining lymph nodes contained a large proportion of CD27-negative and presumably IL-17-biased cells comprising 70–80% of the cells (Figure 3B left). However, in NOD mice, CD27- cells were less frequent within the already smaller V**γ**4+ population (Figure 3B left), and the absolute numbers of CD27-V**γ**4+ cells obtained from skin-draining lymph nodes were much lower, representing a tenth or less of the number in matched B6 controls (Figure 3B right). Further characterization nonetheless showed that in NOD as in B6 mice, a majority of these CD27-V**γ**4+ skin-draining lymph node cells expressed both CCR6 and high levels of CD44 (CD44-hi), further indications of an IL-17 bias [38], whereas only a minority of the V**γ**4+ CD27- cells in spleen and even fewer in pancreatic lymph nodes of either strain were both CCR6+ and CD44-hi (Figure S2). NOD mice also had somewhat smaller numbers of the CD27+V**γ**4+ (IFN**γ**-biased) subpopulation in skin-draining lymph nodes than B6 mice (Figure 3C). Clearly, the paucity of V**γ**4+ **γδ** T cells in skin-draining lymph nodes, and particularly of those biased to produce IL-17, is a distinguishing feature of the **γδ** T cell repertoire in the NOD strain.

### 3.4. Intestinal Intraepithelial Lymphocyte (IEL) γδ T Cells Differ in CD8α Expression in NOD vs. B6 Mice

**Figure 4:**
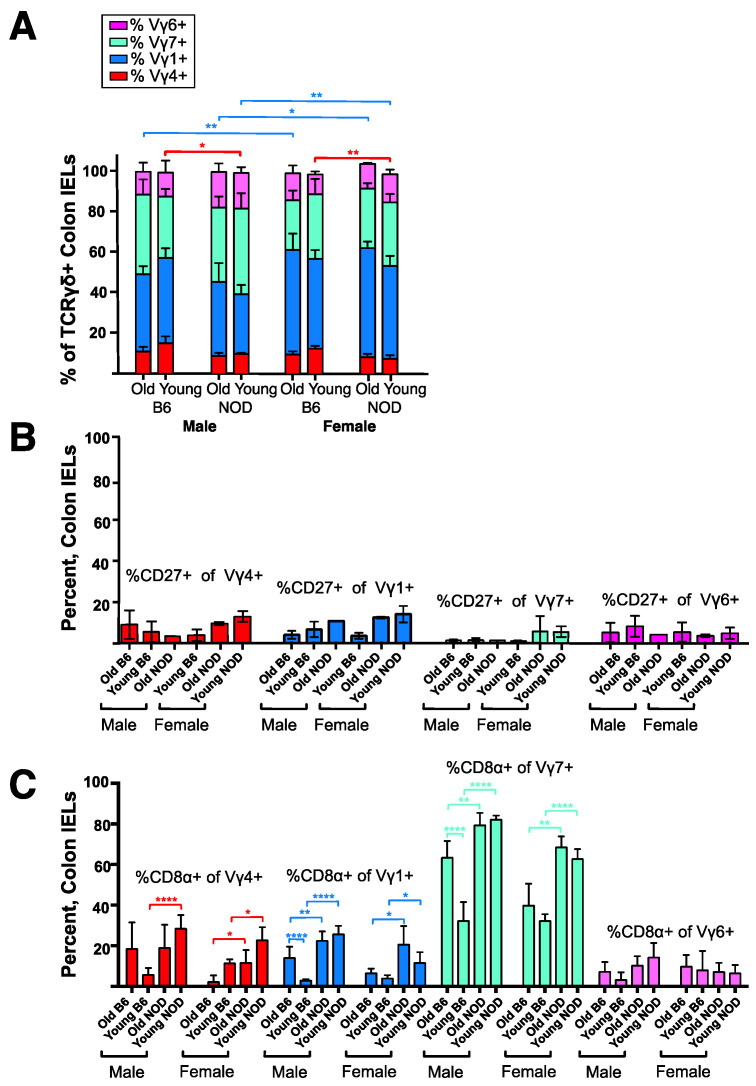
Comparison of colon intraepithelial lymphocyte (IEL) derived T cells in NOD vs. B6 mice. Results for samples from 3–7 mice per group analyzed by flow cytometry, except for C in which only 2 old B6 males, 2 old NOD females, and 1 old NOD male were available for the analysis (errors bars for old B6 male and old NOD female groups show the range obtained rather than the sample standard deviation). (**A**). The mean percentage of each of four V**γ**-defined **γδ** T cell subsets as a fraction of all **γδ** TCR+ cells, among the IELs obtained from individual colons. (**B**). Within each group, the mean percentage of CD27+ cells was relatively low for colon IELs of the V**γ**1, V**γ**4, V**γ**7, and V**γ**6 subsets. Scale matches that used in C. (**C**). Within each group, the mean percentage of CD8α+ cells for colon IELs of the V**γ**1, V**γ**4, V**γ**7, and V**γ**6 subsets was generally higher in NOD mice than in matched B6 controls. * *p* < 0.05, ** *p* < 0.01, and **** *p* < 0.0001.

Taking into account the prominence of **γδ** T cells in the intestinal epithelium, and reported connections between diabetes in humans or NOD mice and the composition of the gut microbiota [48,49,50], we also characterized **γδ** T cells among colon IELs of NOD vs. B6 mice. The data are shown in Figure 4 and Figure S3. Despite the differences evident in spleen and skin-draining lymph nodes, the composition of colon **γδ** T IELs of NOD mice largely resembled those of B6 (Figure 4A), although the percentage of **γδ** T cells varied considerably between individuals (Figure S3A). Due to the low numbers of IELs obtained per mouse, we could not directly test colon IEL V**γ**4+ cells for their bias to produce IL-17. Although the majority were CD27-, this might not be predictive of an IL-17 bias for IEL V**γ**4+ cells, since CD27 expression, though common in spleen, was in any case very low among IELs for all **γδ** T cell subsets (Figure 4B). In contrast, larger fractions and sometimes the majority of the CD4+ and CD8+ **γδ** IELs were CD27+ (Figure S3B). The NKT-like V**γ**1V**δ**6.3+ subpopulation in contrast was less common among colon IELs than in lymphoid organs (see Figure 2A left above), representing only 5–10% in all groups (Figure S3C). Gut **γδ** T cells have been previously noted to be distinct in that a high proportion express the CD8α homodimer (reviewed in [51]). Except for V**γ**6+ cells, we found that almost all other **γδ** IEL in NOD mice expressed CD8α at significantly higher frequencies than their B6 counterparts, especially the V**γ**7+ IELs in NOD males in which about 80% were CD8α+ (Figure 4C). This was the only clear difference among colon IELs that we observed between the two strains.

### 3.5. NOD Mice Lacking Vγ1+ Cells Are Less Prone to Develop Diabetes, Whereas Those Deficient in Vγ4+ Cells Develop Accelerated Disease

**Figure 5:**
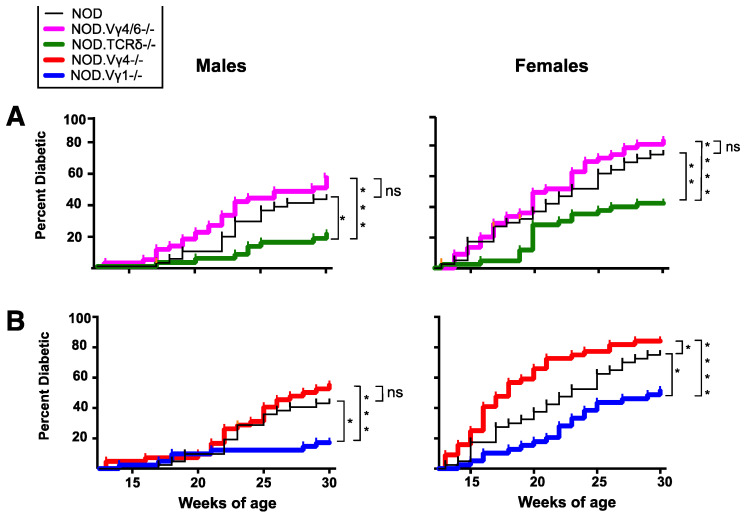
The incidence of spontaneous diabetes in **γδ** T cell subset-deficient NOD mice. Mice were monitored weekly for non-fasting blood glucose levels. Kaplan–Meier plots for each strain show the percent of the mice that had tested positive for diabetes by the indicated week of age, tested between 13–30 weeks of age, for males (left) and females (right). For each group, 38–46 mice were analyzed. (**A**). Results from male and female NOD.TCR**δ**-/- mice and NOD.V**γ**4/6-/- mice juxtaposed to those from wt NOD controls during a similar time period. (**B**). Results from male and female NOD.V**γ**4-/- and NOD.V**γ**1-/- mice juxtaposed to those from the wt NOD controls during a similar time period. * *p* < 0.05, ** *p* < 0.01, *** *p* < 0.001, **** *p* < 0.0001, ns: not significant.

Because differences in the **γδ** T cell compartments of B6 mice and NOD mice were already evident in young mice prior to the onset of diabetes, it seemed possible that **γδ** T cells could influence disease development. The two major **γδ** T cell subsets that were different in the lymphoid organs of NOD mice, V**γ**1+ and V**γ**4+ **γδ** T cell subsets, had been shown to affect pathogenesis in other mouse models of disease [8,9,10,12,15,16,17,52,53,54]. We therefore went on to investigate these two subsets in subsequent functional studies. Figure 5 shows the development of diabetes in **γδ** T cell-deficient NOD mice as they age, compared to age- and sex-matched normal NOD controls, for males and females. The NOD **γδ** T cell-deficient strains were generated by backcrossing gene-targeted mice of the C57BL/6J (B6) background onto the NOD background, except for the NOD.V**γ**4-/- strain, which was generated directly in NOD embryos using CRISPR/Cas9 technology. We monitored mice lacking all **γδ** T cells or particular **γδ** T cell subsets (defined by expression of certain V**γ** genes) weekly from 13 until 30 weeks of age. Both male and female NOD mice deficient in all **γδ** T cells (NOD.TCR**δ**-/- mice) exhibited a substantial decrease in diabetes incidence (Figure 5A), that was less pronounced than but consistent with that seen in an earlier report [7]. NOD.V**γ**1-/- mice showed a similar decrease, such that only ~50% of the females and ~20% of the males developed diabetes by 30 weeks of age, compared to ~75% and 40% in matched wt female and male controls, respectively (Figure 5B). The fact that the absence of V**γ**1+ cells essentially phenocopied the absence of all **γδ** T cells suggested that the disease-promoting **γδ** T cells are within the V**γ**1+ subset, and perhaps only there. In marked contrast, NOD.V**γ**4/6-/- mice (lacking both V**γ**4+ and V**γ**6+ **γδ** T cells), did not differ significantly from normal controls in disease incidence (Figure 5A), emphasizing a unique role for V**γ**1+ **γδ** T cells in promoting NOD diabetes. However, they showed a slight increase in disease incidence, which could suggest that protective **γδ** T cells also exist.

To better reveal potential protectors, we therefore generated a new mouse strain, NOD.V**γ**4-/- mice, using CRISPR/Cas9 technology, lacking V**γ**4+ cells but still capable of producing the V**γ**6+ subset. NOD.V**γ**4-/- females showed a clear acceleration in the development of diabetes, such that by 21 weeks of age their overall incidence rose to ~75%, a level reached by wt females only at ~30 weeks of age (Figure 5B right). Diabetes incidence at the final timepoint was also somewhat higher for the NOD.V**γ**4-/- females than for normal female NOD controls (84% vs. 74%). This suggests that the V**γ**4+ **γδ** T cell population contains protective cells, at least in females, since their absence led to disease acceleration and an increase in diabetes incidence. Disease development in male NOD.V**γ**4-/- mice did not differ significantly from male wt controls (Figure 5B left).

### 3.6. Vγ4+ γδ T Cells in NOD Mice May Provide Protection by Promoting CD4+ Tregs

**Figure 6:**
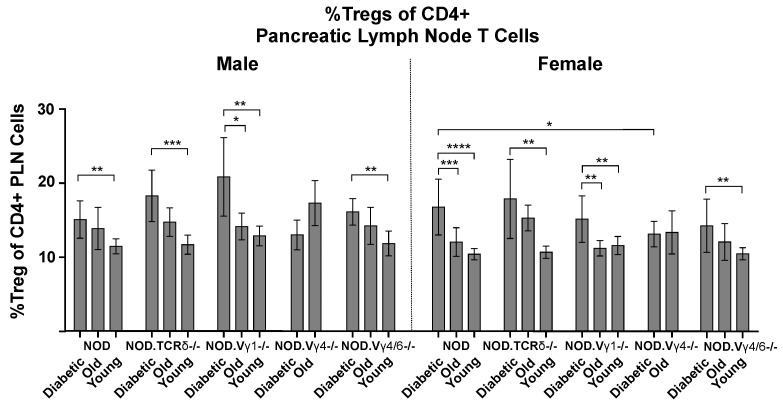
The mean percentage of Tregs present among CD4+ αβ T cells in the pancreatic lymph nodes of NOD-background **γδ** T cell subset-deficient vs. wt NOD mice. Results from mice with recent-onset diabetes were compared to both young and older sex-matched mice, within and between strains. For each group, samples from 3–16 mice were analyzed by flow cytometry, except for two groups, diabetic male NOD.V**γ**1-/- mice and diabetic male NOD.TCR**δ**-/- mice, in which diabetes generally developed very rarely and only 2 mice were available (errors bars for these 2 groups show the range obtained rather than the sample standard deviation). * *p* < 0.05, ** *p* < 0.01, *** *p* < 0.001, and **** *p* < 0.0001.

With this evidence that individual **γδ** T cell subsets play a regulatory role in the development of NOD diabetes, we wondered if they might exert their influence through the more numerous αβ T cells. We first examined the effect of **γδ** T cell ablation on the overall composition of αβ T cells in young, older and diabetic NOD background mice, but found a difference only in NOD.TCR**δ**-/- mice, which showed a high ratio of CD4:CD8 αβ T cells due to a significant decrease in CD8+ αβ T cells (Figure S4A,B). We then looked at regulatory T cells, because other laboratories previously reported an increase in CD4+ CD25+ FoxP3+ “Tregs” in NOD mice as diabetes developed [55,56]. Consistently, though often quite variable, we saw a higher percentage of Tregs in the pancreatic lymph nodes of NOD mice following the onset of diabetes, compared to young normo-glycemic sex-matched NOD controls (Figure 6). In **γδ** T cell subset-deficient NOD mice, we also found higher levels of Tregs among the diabetic mice in the two strains having decreased diabetes susceptibility: the NOD.TCR**δ**-/- and NOD.V**γ**1-/- mice. The NOD.V**γ**4/6-/- mice, whose susceptibility resembled that of wt NOD controls, also showed this increase. In contrast, NOD.V**γ**4-/- mice, of which the females exhibited increased hyperglycemia and accelerated disease development, showed no increase in the pancreatic lymph node Treg percentage with recent onset diabetes (Figure 6). These results suggest that V**γ**4+ **γδ** T cells play a role in bringing about the rise in pancreas-associated Tregs that occurs as diabetes develops, and that this inhibits or slows the development of disease. The balance between V**γ**4+ and V**γ**1+ **γδ** T cells may therefore be critical in disease progression.

### 3.7. Changes in NOD γδ T Cell Populations Associated with the Development of Diabetes, and Evidence for Crosstalk between γδ T Cell Subsets

**Figure 7:**
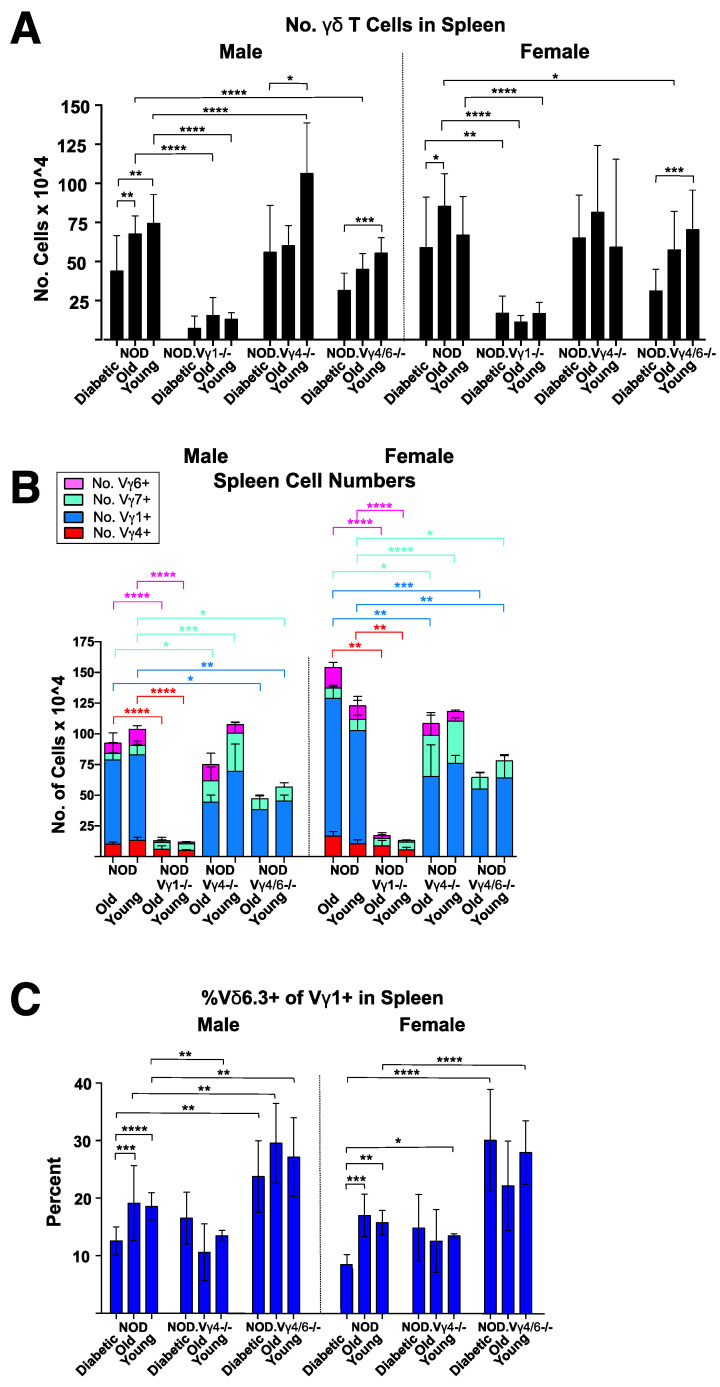
Analysis of **γδ** in the spleens of NOD-V**γ**-deficient mouse strains, compared to those of wt NOD controls. See Figure 6 legend for description of mice used. (**A**). Average number of **γδ** T cells obtained per spleen. (**B**). The average number of cells obtained of each V**γ**-defined **γδ** T cell subset from spleens. (**C**). Mean percentage of spleen V**γ**1+ **γδ** T cells that co-expressed V**δ**6.3. * *p* < 0.05, ** *p* < 0.01, *** *p* < 0.001, and **** *p* < 0.0001.

The number of **γδ** T cells in the spleen of NOD mice has been previously shown to drop with the onset of diabetes [6]. We confirmed this in the spleens of NOD mice with recent onset diabetes, compared to sex-matched older and/or younger NOD mice (Figure 7A). A similar drop in **γδ** T cell numbers was also seen in the spleens of NOD.V**γ**4/6-/- mice that had recently developed diabetes (Figure 7A), suggesting that the decrease primarily if not exclusively involves V**γ**1+ **γδ** T cells. We previously found that V**γ**1+ **γδ** T cells infiltrate the pancreas of NOD mice with insulitis [57], which might explain their depletion in the spleen.

The numbers of cells representing major **γδ** T cell subsets (V**γ**1+, 4+, 6+ and 7+ cells) in the spleen of young and older healthy wt and **γδ** T cell-subset-deficient NOD mice were then compared (Figure 7B), as well as the percent of each **γδ** T cell subset present in spleen, skin-draining and pancreatic lymph nodes (Figure S5). Whether or not they developed diabetes, NOD.V**γ**1-/- mice had much-reduced overall numbers of splenic **γδ** T cells (Figure 7A), indicating that their other **γδ** T cell subsets did not at any stage expand to compensate for the absence of V**γ**1+ cells. NOD.V**γ**4-/- males had normal or slightly increased numbers of splenic **γδ** T cells (Figure 7A) which were mainly V**γ**1+ cells (Figure 7B), perhaps indicating that V**γ**4+ **γδ** T cells normally exert a weak inhibitory effect on their V**γ**1+ counterparts. Compared to normal NOD controls, in NOD.V**γ**1-/- mice, V**γ**4+ cells as expected represented an increased percentage in all lymphoid tissues (Figure S5), as we saw previously in B6.V**γ**1-/- spleens [52]. In both skin-draining and pancreatic lymph nodes, a similar increase in percentage (~2–3-fold) was also seen for the V**γ**6+ and V**γ**7+ subsets of NOD.V**γ**1-/- mice (Figure S5, center and bottom). Oddly, however, in the spleens, the V**γ**6+ percentage did not change, but instead the V**γ**7+ percentage was elevated by 6–8-fold compared to normal controls (Figure S5 top). NOD.V**γ**4-/- mice in contrast had largely unaltered V**γ**1+ and V**γ**6+ percentages in all three lymphoid tissues, but their V**γ**7+ fraction was substantially elevated (Figure S5). NOD.V**γ**4/6-/- mice showed yet another pattern, and had increased percentages of both V**γ**1+ and V**γ**7+ cells in all 3 lymphoid tissues (Figure S5), although the V**γ**1+ fraction was not increased to the degree that we had noted previously in B6.V**γ**4/6-/- spleens [52]. Thus, elimination of particular **γδ** T cell subsets by genetic ablation differentially altered the size of the remaining **γδ** T cell subsets, depending on which subsets were ablated, suggesting that cross-talk between **γδ** T cell subsets affects their survival and/or expansion.

In wt NOD mice with recent onset diabetes, but not in nondiabetic older NOD mice, we noticed a substantial decrease in the fraction of splenic V**γ**1+ cells that express V**δ**6.3 (which include the NKT-like **γδ** T cells; Figure 7C). Their actual numbers would be even more reduced because their total spleen **γδ** T cells numbers also dropped following diabetes onset (Figure 7A). This suggests a distinct role for the V**γ**1+ NKT-like **γδ** T cells in the pathogenesis of NOD diabetes. Intriguingly, in the NOD.V**γ**4/6-/- and NOD.V**γ**4-/- mice (both of which retain V**γ**1+ cells), no selective decrease in the NKT-like V**γ**1+ **γδ** T cells was seen following diabetes onset (Figure 7C), indicating that V**γ**4+ and perhaps V**γ**6+ **γδ** T cells as well may normally regulate V**γ**1+V**δ**6.3+ **γδ** T cell levels. In support of this, NOD.V**γ**4/6-/- spleens contained at all stages an increased percentage of V**γ**1+ cells co-expressing V**δ**6.3 (Figure 7B), as we previously reported in B6.V**γ**4/6-/- mice [52].

**Figure 8:**
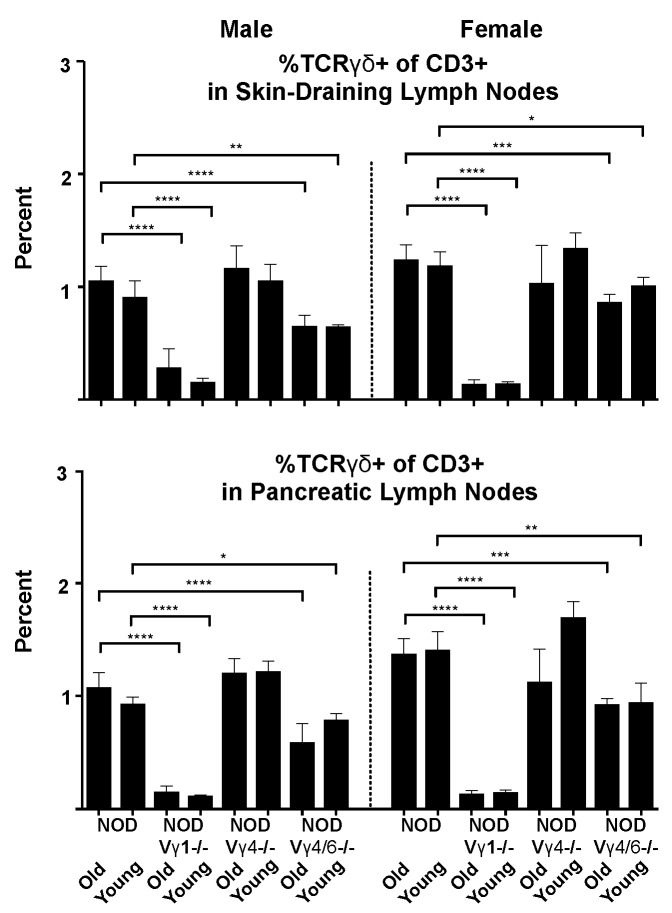
The **γδ** T cell percentage in skin draining and pancreatic lymph nodes of NOD-background **γδ** T cell subset-deficient vs. wt NOD mice. For each group, results of samples from 3–12 mice were analyzed. Results from skin-draining lymph nodes (**top**) and pancreatic lymph nodes (**bottom**) are shown. * *p* < 0.05, ** *p* < 0.01, *** *p* < 0.001, and **** *p* < 0.0001.

In lymph nodes (skin-draining and pancreatic), genetic inactivation of the V**γ**1 gene reduced the frequency of **γδ** T cells among total T cells even more than in the spleen (Figure 8). In contrast, NOD.V**γ**4-/- mice had a normal **γδ** T cell percentage in both lymph node types, and that of NOD.V**γ**4/6-/- mice was only slightly reduced.

**Figure 9:**
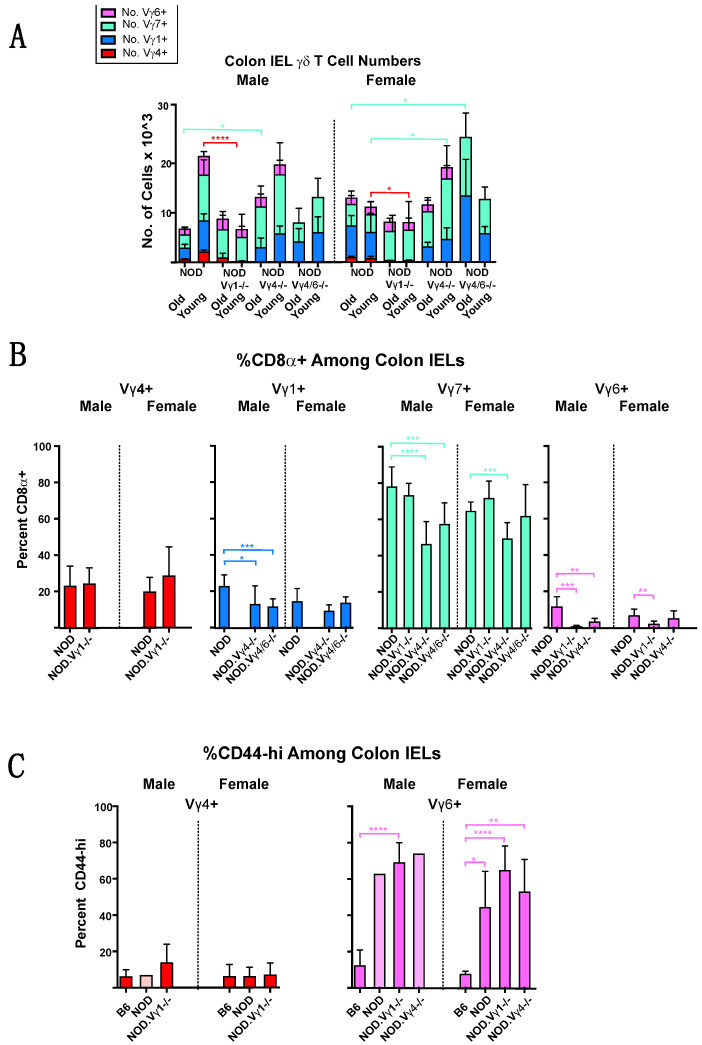
The **γδ** cells remaining in NOD-background **γδ** T cell subset-deficient vs. wt NOD mice among colon IELs. For (**A**–**C**), samples from 3–14 individual mice were analyzed per group, except in C where only a single male NOD and a single male NOD.V**γ**4-/- mouse were available for the analysis. (**A**). The average number of cells obtained for each V**γ**-defined **γδ** T cell subset, in colon IELs. (**B**). The mean percentage of CD8α+ cells in four V**γ**-defined **γδ** T cell subsets, among colon IELs. (**C**). Mean percentages of colon V**γ**4+ IELs (left) and colon V**γ**6+ IELs that express high levels of CD44. Bars in a lighter hue denote data from a single mouse, and are shown for comparison. * *p* < 0.05, ** *p* < 0.01, *** *p* < 0.001, and **** *p* < 0.0001.

Finally, we also examined colon IEL **γδ** T cells in the NOD **γδ** T cell-deficient mouse strains, in young and older nondiabetic mice. We included this experiment because the **γδ** T cell repertoire among IELs is distinct, and genetic ablation of particular **γδ** T cell subsets might in this setting result in unique compensatory effects on other **γδ** T cell subsets that could as well influence the development of diabetes. However, though variability between individuals was high, patterns seen in the spleen and lymph node largely re-emerged in the colon IELs: in NOD.V**γ**1-/- mice, **γδ** T cell numbers overall were lower (although to a lesser degree than in spleen), whereas the other mutant strains had equal or higher numbers of **γδ** T cells than wt (Figure 8). In strains that retained V**γ**1+ cells, V**γ**7+ and V**γ**1+ **γδ** T cells remained the largest IEL subsets (Figure 9A). The V**γ**4+ subset, rarer in any case among the IELs of normal NOD mice, was even further diminished or absent in the colon IELs of NOD.V**γ**1-/- mice (Figure 9A).

However, we did find colon-unique compensatory effects when we examined CD8α expression by **γδ** T IELs (Figure 9B). Here, in both wt and **γδ** T cell-deficient NOD mice, CD8α expression among colon **γδ** IELs was least frequent for V**γ**6+ cells and most frequent for V**γ**7+ cells (results for samples from each sex of each strain are presented here as a single group without further division into old and young segments, as very similar percentages were obtained for both ages). However, the percent of V**γ**7+ cells expressing CD8α was much reduced in NOD.V**γ**4-/- mice and somewhat also in male NOD.V**γ**4/6-/- mice. A similar decrease was seen for CD8α expression on both V**γ**1+ and V**γ**6+ cells as well in the NOD.V**γ**4-/- mice (Figure 9B), perhaps suggesting that V**γ**4+ **γδ** T cells normally promote the survival or expansion of gut CD8α+ **γδ** T cells, especially those of the V**γ**7+ subset.

Lastly, we asked whether either the V**γ**4+ or V**γ**6+ colon IELs were likely to be producers IL-17 (Figure 9C) by looking for high expression of CD44 (CD44-hi), which correlates with an IL-17 bias in these two subsets [47,58,59]. We found very few CD44-hi V**γ**4+ cells in either NOD or NOD.V**γ**1-/- mice. In marked contrast, for colonic V**γ**6+ IELs from wt NOD, NOD.V**γ**1-/- and NOD.V**γ**4-/- mice, half or more were CD44hi, whereas in B6 mice they were much rarer and averaged 20% or less of the V**γ**6+ cells (Figure 9C).

## 4. Discussion

### 4.1. Protective γδ T Cells in NOD Diabetes

Overall, our results indicate that NOD V**γ**4+ **γδ** T cells inhibit the development of diabetes, and suggest that the process by which they do so involves IL-17 production and/or promotion of regulatory CD4+ αβ T cell (Treg) development in the pancreatic lymph nodes. Consistently, in a previous publication using a type 1 diabetes disease model involving adoptive transfer of diabetogenic T cells into NOD/SCID mice, the co-transfer of normal splenic **γδ** T cells capable of producing IL-17 was found to reduce disease incidence [6]. Although IL-17 is generally considered to be a pro-inflammatory cytokine, in several studies IL-17 appeared to suppress type 1 diabetes [48,60,61,62]. Furthermore, in other disease models, IL-17+ **γδ** T cells also have been shown to co-produce amphiregulin [63,64,65], a cytokine which promotes Treg development [66]. That IL-17+ V**γ**4+ cells might protect by increasing Treg levels is plausible because deliberate stimulation of Tregs in NOD mice decreased the development of diabetes [67]. Moreover, **γδ** T cells have been shown to increase levels of Tregs in other autoimmune disease models, including inflammatory bowel disease [68], and autoimmune keratitis [69]. Here, in wt NOD mice, we indeed found that an increase in the percentage of CD4+ Tregs in the pancreatic lymph nodes follows diabetes onset (see Figure 6). Though this increase also occurred in the **γδ** T cell-deficient NOD strains having normal or reduced diabetes susceptibility, it was not found in NOD.V**γ**4-/- female mice which exhibit accelerated diabetes (see Figure 5), consistent with the notion that V**γ**4+ cells normally promote Tregs. If they do, perhaps hyaluronan, which accumulates in the pancreatic islets during type 1 diabetes in humans and in NOD mice and is known to promote the disease [70,71,72], is involved in the mechanism by which the IL-17-biased V**γ**4+ cells are recruited and activated. Due to their proclivity to express high levels of CD44, for which hyaluronan is a known ligand [70], the binding of hyaluronan to CD44 could promote V**γ**4+ IL-17+ T cell activation, as it does for Th17 αβ cells [73], and in this way promote increased survival of Tregs.

Why NOD.V**γ**4/6-/- mice which also lack V**γ**4+ cells did not also fail to expand Tregs upon diabetes onset remains unclear. However, unlike the NOD.V**γ**4-/- mice, NOD.V**γ**4/6-/- mice also have more V**γ**1V**δ**6.3+ cells, a subpopulation that in normal NOD spleens drops upon diabetes onset (see Figure 8). Perhaps the increase in these NKT-like **γδ** T cells in the NOD.V**γ**4/6-/- strain causes Treg cells to expand, as TCR-αβ+ iNKT cells have previously been shown to do [reviewed in [74]].

Why the NOD.V**γ**4-/- males do not like the females show exacerbated diabetes is also puzzling. Since the gut flora of wt male and female NOD mice differs and has been shown to be sufficient to alter their susceptibility to diabetes [49], we speculate that male vs. female intestinal microbiome differences may be responsible. This likely explained a similar scenario in a previous study, where the presence of segmented filamentous gut bacteria correlated with a reduced diabetes incidence in NOD females but not in males, even though the CD4+ Th17 cells stimulated by this organism and thought to be responsible for the decrease were similarly elevated in both sexes [48].

### 4.2. Pathogenic γδ T Cells in NOD Diabetes

In contrast to the protective role of the V**γ**4+ cells, the NOD V**γ**1+ cells appear to promote diabetes development. In a previous study on the role of **γδ** T cells in spontaneous diabetes [7], a larger reduction in the disease incidence of female NOD.TCR**δ**-/- mice was found, implying that the majority of **γδ** T cells promote diabetes. Here, we corroborated and extended this finding by showing reduced diabetes in both female and male NOD.TCR**δ**-/- mice independently backcrossed in our own facility [7]. Our finding that NOD.V**γ**1-/- mice have a reduced incidence of diabetes is consistent with their result, since the vast majority of **γδ** T cells in the lymphoid organs of NOD mice are V**γ**1+ (see Figure 1), though we also found a minor V**γ**4+ population that instead has a protective effect. Indeed, we obtained very similar Kaplan–Meier curves for disease incidence when comparing NOD.TCR**δ**-/- mice to NOD.V**γ**1-/- mice, for both sexes (see Figure 5). Why **γδ** T cell elimination had a somewhat weaker effect on the NOD.TCR**δ**-/- females in our study is unclear. It could be the result of insufficient backcrossing, although only very small amounts of residual non-NOD DNA, not linked to any known insulin-dependent diabetes risk-promoting (*idd*) loci [2,75], remained in our NOD.TCR**δ**-/- mice (see Supp. Detailed Materials and Methods, Genetic Screening), as for the NOD.TCR**δ**-/- mice used in the previous study [7]. However, environmental differences between the mouse colonies may have affected the disease incidence.

Many more V**γ**1+ cells biased to produce IFN**γ** are present in NOD than in B6 mice (see Figure 2D). Given that both sexes of NOD mice lacking V**γ**1+ cells had a reduced incidence of diabetes, our discovery of relatively large numbers of IFN**γ**-biased V**γ**1+ cells in normal NOD mice suggests that these cells intensify the inflammation, and thereby exacerbate on-going autoaggressive attack on the pancreatic islets and the eventual development of diabetes. However, the NOD. V**γ**1-/- mice not only lack V**γ**1+ cells but also showed large decreases in the numbers of **γδ** T cells of all remaining subsets, except for V**γ**7+ cells (see Figure 7B). For this reason, the reduced disease susceptibility in this strain could instead or as well be a consequence of secondary changes in other **γδ** T cells, rather than solely to the absence of V**γ**1+ cells.

We previously reported **γδ** T cell hybridomas generated from NOD-background mice that respond to an insulin-B chain peptide, B:9-23 [76], a major autoantigen in both mouse and human type 1 diabetes that is also recognized by diabetogenic αβ T cells and B cells [77]. Hybridomas expressing both V**γ**4+ and V**γ**1+ TCRs were included among the insulin-peptide reactive NOD-background cells [77]. We also detected V**γ**1+ cells within NOD islets in the pancreas at early stages of insulitis [57]. Thus, **γδ** T cells recruited to the pancreas that respond to insulin peptides could also become specifically activated during the development of diabetes. Several of the B:9-23-responsive **γδ** T cell hybridomas we identified expressed a V**γ**1+V**δ**6.3+ TCR type, and as noted above, the percentage of spleen V**γ**1+ cells co-expressing V**δ**6.3 dropped in NOD mice following recent diabetes onset (see Figure 7C). Perhaps a decrease in this subpopulation in the lymphoid organs occurs because these cells relocate to the pancreas. As noted above, V**γ**1+V**δ**6.3+ cells also represent a previously defined NKT-like subpopulation within the V**γ**1+ subset which has distinct functional properties [33,34,35], and thus they could actually play a different role in diabetes development than do the other V**γ**1+ cells. However, because insulin peptide specificity was not limited to V**γ**1+ **γδ** T cells, it may not be directly related to pathogenicity.

### 4.3. Elimination of Particular γδ T Cell Subsets Precipitates Changes in Other T Cells

Apart from the lack of a Treg increase in NOD.V**γ**4-/- mice following diabetes onset (see Figure 6), and increased prevalence of V**γ**1V**δ**6.3+ cells in NOD.V**γ**4/6-/- mice (see Figure 7C), additional changes in the abundance of other non-targeted T cell types were found in **γδ** T cell-deficient NOD strains. These include a decrease in CD8+ αβ T cell numbers in NOD.TCR**δ**-/- mice (see Figure S4), a reduction in numbers of other non-targeted **γδ** T cell subsets in NOD.V**γ**1-/- spleens (see Figure 7B), and a marked expansion of the V**γ**7+ subset in NOD.V**γ**4-/- mice (see Figure 7B). These findings may suggest that cross-talk occurs between various **γδ** T cell subsets and several other T cell types, including other **γδ** T cells.

In the large intestinal epithelium, a site in which **γδ** T cells are relatively abundant, we found that intraepithelial (IEL) **γδ** T cells of NOD mice for the most part resemble those of B6 mice, with two exceptions. First, NOD **γδ** IELs express CD8α at significantly higher frequencies, particularly within the V**γ**7+ subset, the predominant **γδ** T cell subset found in colon IELs in both strains (see Figure 9B). Second, the majority of NOD-derived V**γ**6+ colon IELs appear to be IL-17 biased because of their CD44-hi phenotype, unlike those of B6 mice See Figure 9C). Insufficient gut CD8α+ V**γ**7+ **γδ** T cell numbers might contribute to the diabetes acceleration, since these cells were decreased in NOD.V**γ**4-/- mice (see Figure 9B), but IL-17-biased V**γ**6+ gut IELs probably do not, because they did not differ substantially from those of wt NOD mice in any of the **γδ** T cell-deficient NOD strains.

### 4.4. The Role of γδ T Cells in Human Type 1 Diabetes

In humans, several studies have shown that **γδ** T cells, including V**γ**9V**δ**2+ **γδ** T cells [78] and CD8+ **γδ** T cells [79,80,81], increase prior to disease onset in diabetes-prone individuals, and decrease with disease exacerbation [78,79,80,81,82,83]. However, whether such observations indicate that **γδ** cells protect against the development of diabetes in humans, or instead that their expansion is merely a passive marker brought about by other processes as diabetes develops, is unclear. Our study provides evidence that in type 1 diabetes in mice, some **γδ** T cells actively promote disease development, while others protect against it. Our results further suggest that **γδ** T cells at least in part influence NOD diabetes by inducing changes in the levels of other T cells, including CD4+ Tregs as well as other **γδ** T cell subsets. In human type 1 diabetes, **γδ** T cells might influence diabetes development in similar ways.

## Data Availability

Not applicable.

## Supplementary Materials

The following supporting information can be downloaded at: https://www.mdpi.com/article/10.3390/biom12101406/s1. Figure S1: Comparison of **γδ** T cell subsets producing IFN**γ**, IL-17, and IL-2 in NOD vs. B6 mice; Figure S2: Identification of IL-17-biased V**γ**4+ cells in NOD vs. B6 mice; Figure S3: Colon IELs from NOD vs. B6 mice; Figure S4: Major T cell populations in NOD-background **γδ** T cell-deficient vs. wt NOD mice in those with recent-onset diabetes vs. nondiabetic old and young mice; Figure S5: The **γδ** T cell repertoire of NOD-background **γδ** T cell subset-deficient vs. wt NOD mice in lymphoid organs; and Detailed Materials and Methods S1 [84,85,86,87,88,89].

## Abbreviations

T cell Receptor: TCR, wild type: wt, intestinal intraepithelial lymphocytes: IELs, CD4+ αβ T regulatory cells: Tregs, CD4+ αβ+ IL-17-biased T-helper: Th17, specific pathogen-free: SPF, single nucleotide polymorphism: SNP, Natural Killer-like T cells: NKT.
