# Supplementary material for: A Distinctive γδ T Cell Repertoire in NOD Mice Weakens Immune Regulation and Favors Diabetic Disease
Source: Biomolecules. 2022 Oct 1;12(10):1406. doi: 10.3390/biom12101406 (PMC9599391; doi:10.3390/biom12101406)
Supplement: Supplementary file 1 [file biomolecules-12-01406-s001.zip › Supplemental materials folder/Detailed Materials and Methods R.pdf]

Detailed Materials and Methods

Mouse Strains. Wildtype (wt) NOD/ShiLtJ (NOD) mice were purchased from Jackson Laboratories (Bar Harbor, ME, USA) and maintained in our colony. NOD.TCR -/- mice, lacking all T cells, were generated from B6.TCR -/- mice (B6.129P2-*Tcrd*^{tm1Mom}/J, Jackson Laboratories, Bar Harbor, ME, USA) that originated from a genetically manipulated 129/Ola strain ES cell line [1,2]. The NOD.TCR -/- strain was established after a total of 10 times backcrosses onto the NOD background, selecting offspring for the inheritance of the *neo*-inactivated C allele via Southern blotting using a *neo* probe, and the final strain then established by intercrossing. The NOD.V 1-/- strain (NOD.129-TcrVg1^{tm1car}) was similarly generated starting with mice originally from a 129/SvJ ES cell line having the V 1 gene inactivated by *neo* gene insertion [3]. Offspring carrying the inactivated V 1 allele were selected via Southern blot analysis using a *neo* probe, and a homozygous strain was established after 10 total backcrosses. The NOD.V 4/6-/- strain (NOD.129-TcrVg4Vg6^{tm1Iku}) was similarly generated starting with B6.V 4/6-/- mice originally from a 129/Ola ES cell line in which the V 4 gene was inactivated by a frame-shift mutation and the V 6 gene was deleted [4]. Offspring carrying the mutant allele were identified via Southern blot analysis using a probe for the nearby V 5 gene to detect the presence of a novel ~5Kb BamHI band, and a homozygous strain was established after 10 backcrosses.

For NOD.V 4-/- mice (NOD.129-TcrVg4^{tm1Mat}), CRISPR/Cas9 technology was used to generate the mutation directly in pure NOD background zygotes. Guide RNA was designed using the CRISPOR design software and guide activity was verified using the Guide It sgRNA screening kit (Clontech, Takara Bio USA, Mountain View, CA, USA). Following in vitro fertilization, zygotes were microinjected with Cas9 mRNA (Sigma-Aldrich Products, St. Louis, MO, USA) plus the following two guide RNAs to target deletion of the entire V 4 gene:

172 for: GACAGCTGAAACTTACGCA CGG

230 for: GAACTTTCCATGCAACGAC TGG

After microinjection, the NOD zygotes were transferred into pseudopregnant recipients. The resulting F0 pups were genotyped by PCR using primers near the V 4 gene to identify putative positive founders, from tail snips taken from the pups at weaning as the DNA source, which confirmed deletion of a ~775 bp segment in this region (including the entire V 4 gene) in at least one allele, in 32 out of 37 total mice. Three primers were used to ascertain this:

V 4 For 1-1: 5′- GTC AGC AAA CCA TTT CAA AGA C-3′

V 4 Del Rev: 5′-CCA ATC ATG CAG TGA GCC TGC -3′

V 4 wt Rev -1-1: 5′-GCA GTC TCA TCT CTT GGT CTG-3′

The V 4 For 1-1 and V 4 Del Rev primers generated a 1139 bp product from the wt V 4 locus and a 364 bp product from the mutated V 4 locus, with the exact product size varying depending upon indels that may be introduced during DNA repair. By contrast, the V 4 For 1-1 and V 4 wt Rev -1-1 primers amplify the unmodified V locus only to generate a 581bp product. PCR conditions used were as follows: Tm 58°C, extension 40 seconds, 35x cycles.

After choosing a founder male with a single deleted V 4 gene, the top 5 predicted off target sites for these guide RNAs were tested by Sanger sequencing of PCR products, but all proved to be unaltered. The founder male was then bred with a NOD female and the progeny subsequently cross-bred to establish the homozygous NOD.V 4-/- strain. All mutant NOD strains, once established, were housed in the same room in the National Jewish Biological Resource Center with unmodified NOD mice and maintained under SPF conditions. All mice were cared for following guidelines for normal and immune-deficient animals, and all experiments conducted as outlined under a protocol approved by the National Jewish Health Institutional Animal Care and Use Committee.

Genetic Screening of Mutant Mouse Strains. For NOD.TCR -/- mice, breeders from backcrosses 2-6 were chosen that had inherited the greatest number of NOD loci (vs. B6 or 129) using “speed congenics” to identify the presence of NOD-derived DNA satellites in PCR-amplified tail snip DNA samples (Barbara Davis Center Molecular Biology Service Center, Aurora, CO, USA). After 4 additional backcrosses, the established NOD.TCR -/- line was tested again against 152 SNPs distributed throughout the genome that are known to differ between NOD and the 129 strain (Jackson Laboratory, Bar Harbor, ME, USA). All SNPs were NOD-derived except for 2 alleles within an 18 Mb segment on both chromosomes 14 (containing the TCR locus); thus, 98.5% of the SNPs tested in the NOD.TCR -/- strain were NOD-derived. In the case of the NOD.V 1-/- and NOD.V 4/6-/-mice, no genetic screening was done until the strains had been established following 10 backcrosses, then each was tested for the sequences of 262 NOD- vs. 129-derived SNPs distributed throughout the genome (Jackson Laboratory, Bar Harbor, ME, USA). For the NOD.V 1-/-mice, all tested SNPs were NOD-derived except for 3 alleles within a 38.6 Mb segment on both chromosomes 13 (containing the TCR locus); thus, 97.73% of the SNPS tested in the NOD.V 1-/- strain were NOD-derived. For the NOD.V 4/6-/-mice, all SNPs were NOD-derived except for 1 allele within a 38.6 Mb segment on both chromosomes 13 (containing the TCR locus); thus, 99.24% of the SNPs tested in the NOD.V 4/6-/-

strain were NOD-derived. No genetic screening was done for the NOD.V 4-/- strain, because the mutation was made directly in NOD zygotes using CRISPR/Cas9, and thus no foreign DNA was introduced except the few bp used to delete the V 4 gene.

Cell Preparation. Spleen and lymph node cells from individual mice were prepared by mechanical dispersion of each organ in Balanced Salts Solution (BSS) [5] plus 5% FBS, by pushing them through Cellector (ThermoFisher Scientific, Waltham, MA, USA) screens using a plunger from a 3 ml syringe. For spleen cells, red blood cells were then lysed using Gey's solution. Pancreatic lymph node cells, usually 2 per mouse, were pooled for each sample, as were skin-draining lymph nodes, generally 4 per mouse, to include 2 inguinal and 2 axillary lymph nodes per animal. Prepared cell suspensions were either held on ice or at 4°C until ready for staining, or were immediately stimulated with PMA/ionomycin to test for the cytokines that were produced (see below). Intestinal intraepithelial lymphocytes (IEL) were prepared from the colons of each mouse based on a previously published protocol [6]. Briefly, intestines were dissected out, then placed in cold PBS in a petrie dish, and any attached mesenteries cut to straighten them out. The colon was separated by cutting just below the caecum, and any obvious blood vessels attached to it were dissected away. Using a syringe and an 18g needle, the colon was then flushed out with fresh cold PBS, and transferred to a clean petrie dish. The intestine was then opened longitudinally with scissors, and snipped into ~1 cm pieces and agitated in the plate to rinse. After transferring the pieces to a 50 ml conical tube, 5 ml of predigestion buffer (BSS plus 6.6 mM EDTA and1 mM dithiothreitol) was added, and the pieces incubated at 37°C for 40 minutes while rotating slowly. After vortexing the tube for about 10 seconds, the pieces were place in a Falcon 70 m cell strainer (ThermoFisher Scientific, Waltham, MA, USA) and the flowthrough collected in a clean 50 ml tube. The pieces were then replaced in the original tube, 5 ml of additional pre-digestion buffer added, and incubated as before for another 40 minutes. After vortexing again, the pieces were once more poured into the cell strainer and the flowthrough added to the previous. Debris in the collected flowthrough was allowed to settle for ~30 min. at 4°C, and the supernatant then transferred to a 15 ml tube. Tubes were centrifuged at 1200 rpm, for 10 minutes, the pellet resuspended in 1 ml BSS + 5% FBS + DNAse (25 l/tube of a 1 mg/ml solution in BSS with 50% glycerol), and incubated for 5 minutes in a 37°C water bath. After re-centrifugation, the cells were resuspended in BSS + 5% FBS.

Flow Cytometry. Cell preparations were surface stained for flow cytometry as previously described [7], in 96-well U-bottom plates with BSS plus 2% FBS and 0.1% sodium azide as staining buffer, and

commercially available fluorochrome labeled monoclonal antibodies. In addition, some monoclonal antibody reagents were prepared in our own laboratory, including biotinylated anti-V 7 [clone GL1.7 [8]], biotin-labeled anti-V 6.3 [clone 17C [9]], FITC-labeled anti-CD4 [clone GK1.5 [10]], and unlabeled anti-Fc R II/III for blocking [clone 2.4G2 [11]]. All samples were analyzed on an LSRII flow cytometer (Becton Dickinson Biosciences, Franklin Lakes, NJ, USA) and the FCS files were processed using FlowJo 9.9 software (TreeStar; Becton Dickinson Biosciences, Franklin Lakes, NJ, USA).

Intracellular cytokine staining. Before staining for flow cytometry, in some experiments, spleen cells were first nonspecifically activated by culturing them in supplemented Iscove's Modified Dulbecco's medium [12] containing 5% FBS, plus Brefeldin A (10   g/ml), PMA (75 ng/ml) and ionomycin (1.6   g/ml), for 4-5 hours at 37°C in air containing 10% CO_2. Following surface staining of the cells, intracellular cytokine staining for IFN , TNF , IL-17, and/or IL-2 was carried out as previously described [13]. Briefly, the stained cells were first fixed by incubating them in 1% paraformaldehyde in PBS at 4°C overnight in the dark. After washing in staining buffer, the cells were permeabilized by incubating them in 0.5% saponin in PBS (SAP buffer) at 4°C for 10 minutes. After centrifugation, the supernatant was removed, and anti-cytokine antibodies diluted in in SAP buffer plus 5   g/ml unlabeled anti-FcR antibody (clone 2.4G2) added. After a 30-minute incubation at 4°C, the cells were washed once in SAP buffer, once in flow cytometry staining buffer, resuspended in 1% paraformaldehyde in PBS, and analyzed by flow cytometry.

Staining for nuclear transcription factors. In some experiments, nuclear staining for transcription factors was carried out, using freshly isolated unstimulated cells, with eBioscience Permeabilization and Fixation/Permeabilization Buffers (ThermoFisher Scientific, Waltham, MA, USA), in accordance with the manufacturer's instructions. Briefly, after surface staining, cells were fixed for 45 minutes at 4°C in Fixation/Permeabilization buffer. After washing in the same buffer, cells were first pre-blocked with 10% rat serum and 40   g/ml hamster IgG in Permeabilization buffer for 15 minutes at room temperature in the dark, and antibodies specific for PZLF, Tbet, and/or FoxP3 then added and the cells incubated for another 30 minutes in the dark at room temperature. After washing twice in Permeabilization Buffer, cells were fixed in 1% paraformaldehyde in PBS and analyzed by flow cytometry.

Reference

1. Itohara, S.; Mombaerts, P.; Lafaille, J.; Iacomini, J.; Nelson, A.; Clarke, A.R.; Hooper, M.L.; Farr, A.; Tonegawa, S. T cell receptor δ gene mutant mice: Independent generation of αβ T cells and programmed rearrangements of γδ TCR genes. *Cell* **1993**, *72*, 337–348.

2. Mombaerts, P.; Arnoldi, J.; Russ, F.; Tonegawa, S.; Kaufmann, S.H.E. Different roles of αβ and γδ T cells in immunity against an intracellular bacterial pathogen. *Nature* **1993**, *365*, 53–56.
3. Andrew, E.M.; Newton, D.J.; Dalton, J.E.; Egan, C.E.; Goodwin, S.J.; Tramonti, D.; Scott, P.; Carding, S.R. Delineation of the function of a major γδ T cell subset during infection. *J. Immunol.* **2005**, *175*, 1741–1750.
4. Sunaga, S.; Maki, K.; Komagata, Y.; Miyazaki, J.-I.; Ikuta, K. Developmentally ordered V-J recombination in mouse T cell receptor γ locus is not perturbed by targeted deletion of the Vγ4 gene. *J. Immunol.* **1997**, *158*, 4223–4228.
5. Mishell, R.I.; Dutton, R.W. Immunization of dissociated spleen cell cultures from normal mice. *J. Exp. Med.* **1967**, *67*, 423–442.
6. Weigmann, B.; Tubbe, I.; Seidel, D.; Nicolaev, A.; Becker, C.; Neurath, M.F. Isolation and subsequent analysis of murine lamina propria mononuclear cells from colonic tissue. *Nat. Protoc.* **2007**, *2*, 2307–2311.
7. Roark, C.L.; French, J.D.; Taylor, M.A.; Bendele, A.M.; Born, W.K.; O'Brien, R.L. Exacerbation of collagen-induced arthritis by oligoclonal, IL-17-producing γδ T cells. *J. Immunol.* **2007**, *179*, 5576–5583.
8. Goodman, T.; Lefrancois, L. Intraepithelial lymphocytes: Anatomical site, not T cell receptor form, dictates phenotype and function. *J. Exp. Med.* **1989**, *170*, 1569–1581.
9. Belles, C.; Kuhl, A.L.; Donoghue, A.J.; Sano, Y.; O'Brien, R.L.; Born, W.; Bottomly, K.; Carding, S.R. Bias in the γδ T cell response to *Listeria monocytogenes*. Vδ6.3$^+$ cells are a major component of the γδ T cell response to *Listeria monocytogenes*. *J. Immunol.* **1996**, *156*, 4280–4289.
10. Dialynas, D.P.; Quan, Z.S.; Wall, K.A.; Pierres, A.; Quintans, J.; Loken, M.R.; Pierres, M.; Fitch, F.W. Characterization of the murine T cell surface molecule, designated L3T4, identified by monoclonal antibody GK1.5: Similarity of L3T4 to the human Leu-3/T4 molecule. *J. Immunol.* **1983**, *131*, 2445–2451.
11. Unkeless, J.C. Characterization of a monoclonal antibody directed against mouse macrophage and lymphocyte Fc receptors. *J. Exp. Med.* **1979**, *150*, 580–588.
12. O'Brien, R.L.; Fu, Y.-X.; Cranfill, R.; Dallas, A.; Reardon, C.; Lang, J.; Carding, S.R.; Kubo, R.; Born, W. Heat shock protein Hsp-60 reactive γδ cells: A large, diversified T lymphocyte subset with highly focused specificity. *Proc. Natl. Acad. Sci. USA* **1992**, *89*, 4348–4352.
13. Roark, C.L.; French, J.D.; Taylor, M.A.; Bendele, A.M.; Born, W.K.; O'Brien, R.L. Exacerbation of collagen-induced arthritis by oligoclonal, IL-17-producing γδ T cells. *J. Immunol.* **2007**, *179*, 5576–5583.
