# Supplementary material for: A Distinctive γδ T Cell Repertoire in NOD Mice Weakens Immune Regulation and Favors Diabetic Disease
Source: Biomolecules. 2022 Oct 1;12(10):1406. doi: 10.3390/biom12101406 (PMC9599391; doi:10.3390/biom12101406)
Supplement: Supplementary file 1 [file biomolecules-12-01406-s001.zip › Supplemental materials folder/Fig. S1.pdf]

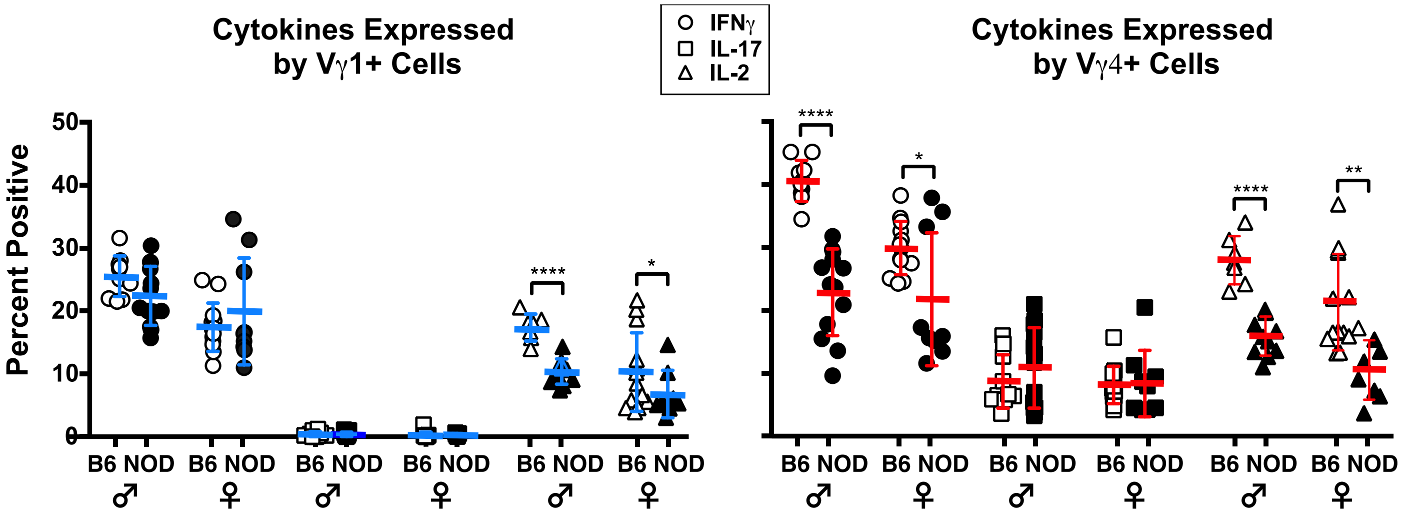

Figure S1. Comparison of γδ T cell subsets producing IFNγ, IL-17, and IL-2 in NOD vs. B6 mice. For each sex-matched group, results of samples from 6-13 mice were analyzed by flow cytometry. Freshly isolated spleen cells were cultured with PMA/ionomycin for ~5 hours before staining. The percent of Vγ1+ cells (left) and Vγ4+ cells (right) expressing a given cytokine is shown for IFNγ (circles), IL-17 (squares), and IL-2 (triangles). Each symbol represents the result from an individual mouse, with horizontal bars showing the mean for each group. Samples from B6 mice are shown as open symbols, and those from NOD mice as filled symbols. * $p < 0.05$, ** $p < 0.01$, and **** $p < 0.0001$.
