# Supplementary material for: A Distinctive γδ T Cell Repertoire in NOD Mice Weakens Immune Regulation and Favors Diabetic Disease
Source: Biomolecules. 2022 Oct 1;12(10):1406. doi: 10.3390/biom12101406 (PMC9599391; doi:10.3390/biom12101406)
Supplement: Supplementary file 1 [file biomolecules-12-01406-s001.zip › Supplemental materials folder/Fig. S2.pdf]

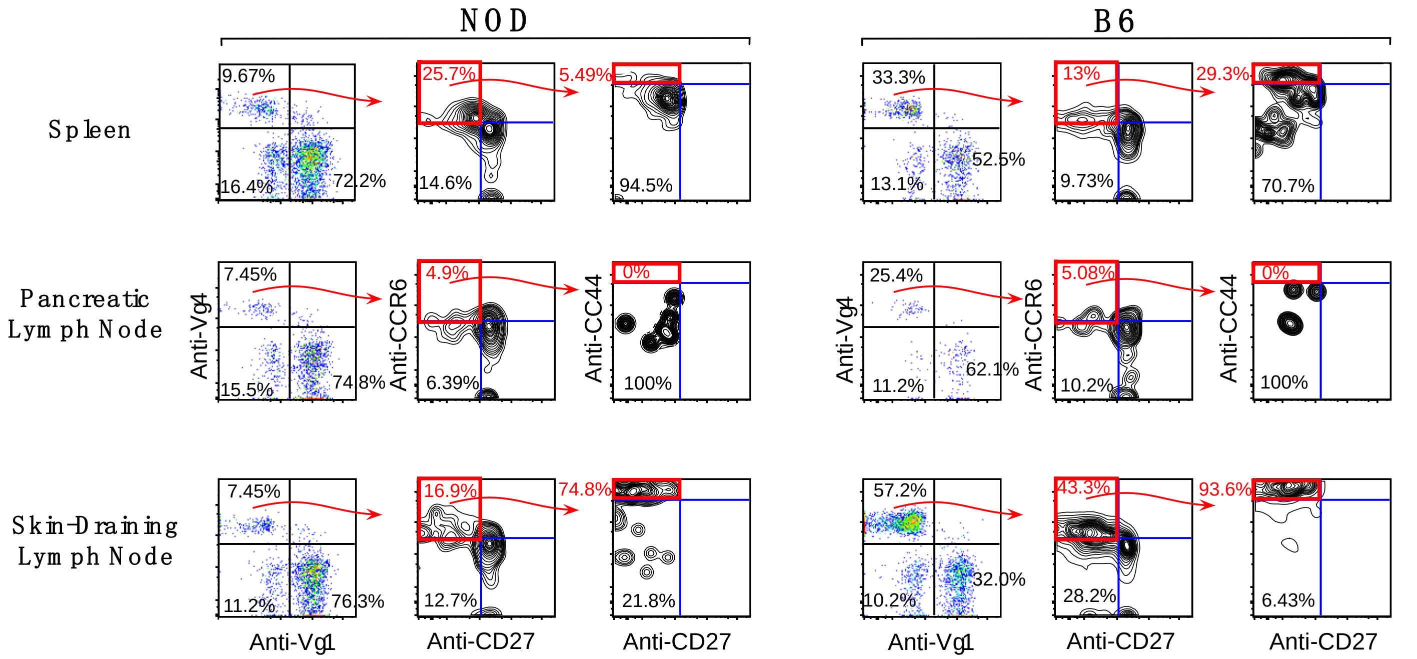

Figure S2. Identification of IL-17-biased Vγ4+ cells in NOD vs. B6 mice. Typical flow cytometry profiles obtained are shown, using freshly isolated cells from a young female mouse of the NOD (left profiles) and B6 (right profiles) strain. Gated CD3+ γδ-TCR+ Vγ1- Vγ4+ -cells (left panels) that, within the CD27-negative Vγ4+ population, were positive for CCR6 (center panels), and expressed high levels of CD44 (CD44hi, right panels), represent likely IL-17-biased cells. Results for cells from spleen (top panels), pancreatic lymph nodes (center panels), and skin-draining lymph nodes (bottom panels) are shown for comparison; the presumably IL-17-biased population in both strains constituted the majority of the Vγ4+ cells in skin-draining lymph nodes, but were less common in spleen and very rare in pancreatic lymph nodes.
