# Supplementary material for: A Distinctive γδ T Cell Repertoire in NOD Mice Weakens Immune Regulation and Favors Diabetic Disease
Source: Biomolecules. 2022 Oct 1;12(10):1406. doi: 10.3390/biom12101406 (PMC9599391; doi:10.3390/biom12101406)
Supplement: Supplementary file 1 [file biomolecules-12-01406-s001.zip › Supplemental materials folder/Fig. S3.pdf]

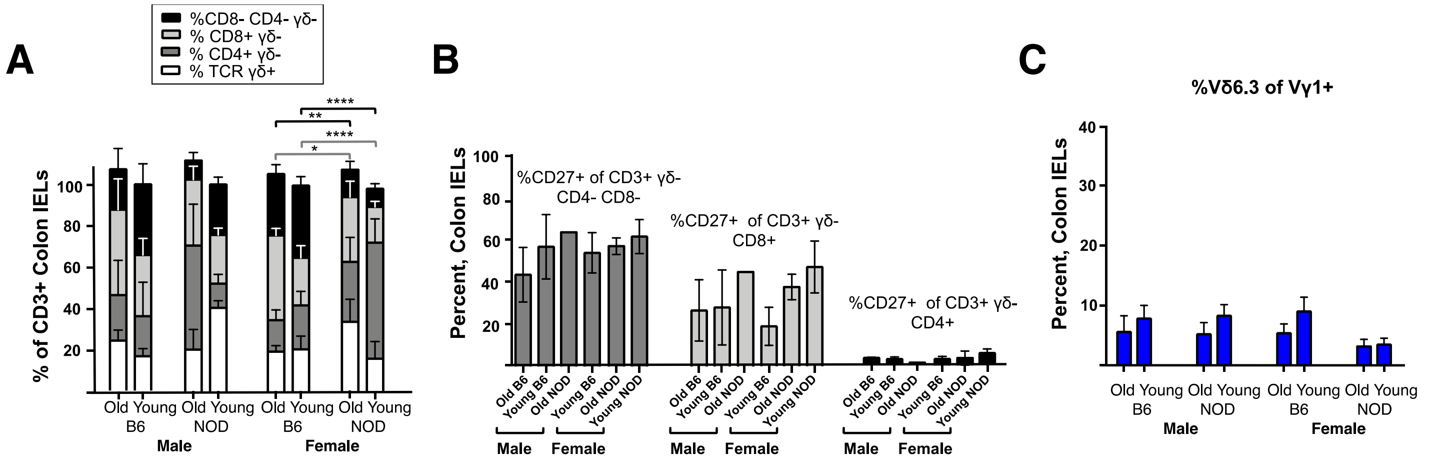

Figure S3 Colon IELs from NOD vs. B6 mice. For each age and sex-matched group shown, results of samples from 3-7 mice were analyzed by flow cytometry, except for C in which only 2 old B6 males, 2 old NOD females, and 1 old NOD male were available for the analysis (errors bars for old B6 males and old NOD females show the range obtained rather than the sample standard deviation). **A.** The mean percentage of colon IELs representing γδ T cells, CD4+ αβ T cells, CD8+ αβ T cells, and CD4-CD8- αβ T cells within each group is shown, as a percentage of all CD3+ cells obtained. **B.** The mean percentage of CD27+ cells within CD4+ αβ T cells, CD8+ αβ T cells, and CD4-CD8- αβ T cells obtained for each group from colon IELs. **C.** The mean percentage of Vγ1+ cells co-expressing Vδ6.3 among colon IELs obtained for each group. Scale matches that used in Fig. 7C. ***** $p < 0.05$, ** $p < 0.01$, and **** $p < 0.0001$.
