# Supplementary material for: A Distinctive γδ T Cell Repertoire in NOD Mice Weakens Immune Regulation and Favors Diabetic Disease
Source: Biomolecules. 2022 Oct 1;12(10):1406. doi: 10.3390/biom12101406 (PMC9599391; doi:10.3390/biom12101406)
Supplement: Supplementary file 1 [file biomolecules-12-01406-s001.zip › Supplemental materials folder/Fig. S4.pdf]

A

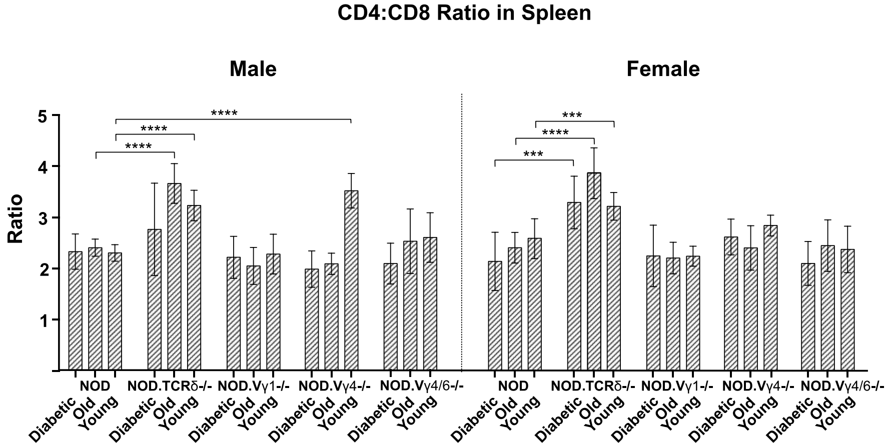

B

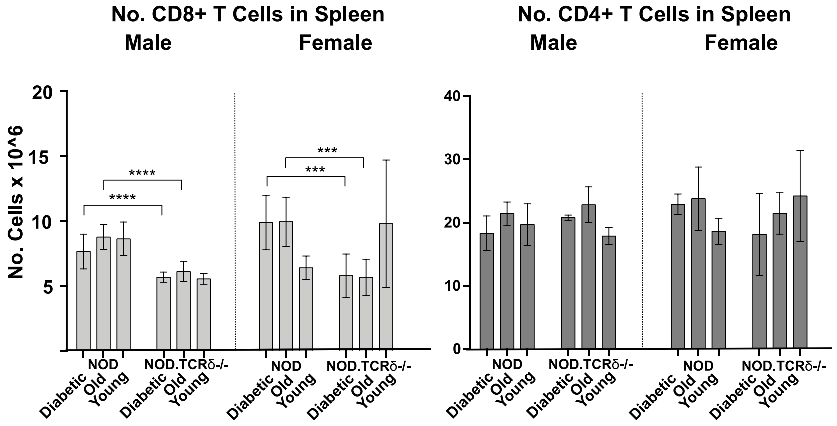

Figure S4. Major T cell populations in NOD-background γδ T cell-deficient vs. wt NOD mice in those with recent-onset diabetes vs. nondiabetic old and young mice. For each group, results of samples from 3-16 mice were analyzed by flow cytometry, except for two groups - diabetic male NOD.Vγ1-/- mice and diabetic male NOD.TCRδ-/- mice - in which diabetes developed very rarely and only 2 mice were available for the analysis (errors bars for the 2 latter groups show the range obtained rather than the sample standard deviation). Sex-matched groups of mice with recent-onset diabetes were compared between strains as well as to sex-matched young and old mice within the same strain. **A.** The mean of the CD4:CD8 ratio in splenic αβ T cells (%CD3+ CD4+ TCRγδ- cells divided by %CD3+ CD8+ TCRγδ- cells). **B.** The average number of CD8+ αβ T cells (left) and CD4+ αβ T cells (right) obtained per spleen for each group.*** $p < 0.001$, and **** $p < 0.0001$
