# Supplementary material for: A Distinctive γδ T Cell Repertoire in NOD Mice Weakens Immune Regulation and Favors Diabetic Disease
Source: Biomolecules. 2022 Oct 1;12(10):1406. doi: 10.3390/biom12101406 (PMC9599391; doi:10.3390/biom12101406)
Supplement: Supplementary file 1 [file biomolecules-12-01406-s001.zip › Supplemental materials folder/Fig. S5.pdf]

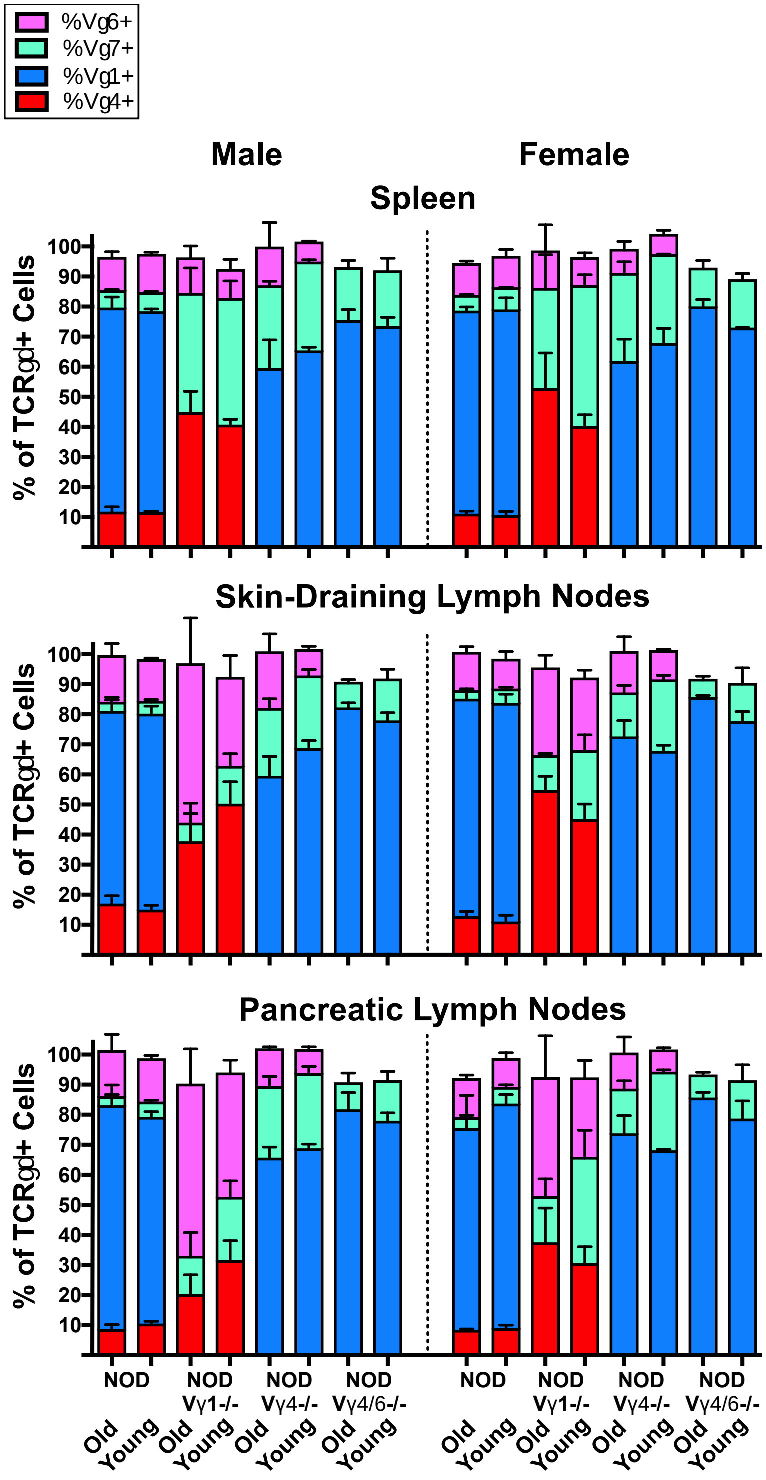

Figure S5. T γδ ell repertoire of NOD-background γδ T cell subset-deficient vs. wt NOD mice in lymphoid organs. Averages of flow cytometry results from cells obtained from individual mice are shown. For each group in A-D, results of samples from 3-12 mice were analyzed. The mean percentage of each Vγ-defined γδ T cell subset is shown as a fraction of all γδ TCR+ cells, in spleen (top), skin-draining lymph nodes (center), and pancreatic lymph nodes (bottom).
